# α-cyanobacteria possessing form IA RuBisCO globally dominate aquatic habitats

**DOI:** 10.1038/s41396-022-01282-z

**Published:** 2022-07-18

**Authors:** Pedro J. Cabello-Yeves, David J. Scanlan, Cristiana Callieri, Antonio Picazo, Lena Schallenberg, Paula Huber, Juan J. Roda-Garcia, Maciej Bartosiewicz, Olga I. Belykh, Irina V. Tikhonova, Alberto Torcello-Requena, Paula Martin De Prado, Andrew D. Millard, Antonio Camacho, Francisco Rodriguez-Valera, Richard J. Puxty

**Affiliations:** 1 Evolutionary Genomics Group, Departamento de Producción Vegetal y Microbiología, Universidad Miguel Hernández, San Juan de Alicante, Alicante, Spain; 2 School of Life Sciences, University of Warwick, Coventry, CV4 7AL UK; 3 National Research Council (CNR), Institute of Water Research (IRSA), Verbania, Italy; 4 Cavanilles Institute of Biodiversity and Evolutionary Biology, University of Valencia, E-46980 Paterna Valencia, Spain; 5 Department of Zoology, University of Otago, Dunedin, New Zealand; 6 Instituto Tecnológico de Chascomús (INTECH), UNSAM-CONICET., Av. Intendente Marino Km 8,200, 7130 Chascomús, Buenos Aires Argentina; 7 Instituto Nacional de Limnología (INALI), CONICET-UNL., Ciudad Universitaria—Paraje el Pozo s/n, 3000 Santa Fé, Argentina; 8 Department of Environmental Sciences, University of Basel, Basel, Switzerland; 9 Limnological Institute, Russian Academy of Sciences, P.O. Box 278, 664033 Irkutsk, Russia; 10 Department of Genetics and Genome Biology, University of Leicester, Leicester, LE1 7RH UK; 11 Moscow Institute of Physics and Technology, 141701 Dolgoprudny, Russia

**Keywords:** Water microbiology, Microbial ecology, Limnology, Comparative genomics

## Abstract

RuBisCO (ribulose 1,5-bisphosphate carboxylase/oxygenase) is one the most abundant enzymes on Earth. Virtually all food webs depend on its activity to supply fixed carbon. In aerobic environments, RuBisCO struggles to distinguish efficiently between CO_2_ and O_2_. To compensate, organisms have evolved convergent solutions to concentrate CO_2_ around the active site. The genetic engineering of such inorganic carbon concentrating mechanisms (CCMs) into plants could help facilitate future global food security for humankind. In bacteria, the carboxysome represents one such CCM component, of which two independent forms exist: α and β. Cyanobacteria are important players in the planet’s carbon cycle and the vast majority of the phylum possess a β-carboxysome, including most cyanobacteria used as laboratory models. The exceptions are the exclusively marine *Prochlorococcus* and *Synechococcus* that numerically dominate open ocean systems. However, the reason why marine systems favor an α-form is currently unknown. Here, we report the genomes of 58 cyanobacteria, closely related to marine *Synechococcus* that were isolated from freshwater lakes across the globe. We find all these isolates possess α-carboxysomes accompanied by a form 1A RuBisCO. Moreover, we demonstrate α-cyanobacteria dominate freshwater lakes worldwide. Hence, the paradigm of a separation in carboxysome type across the salinity divide does not hold true, and instead the α-form dominates all aquatic systems. We thus question the relevance of β-cyanobacteria as models for aquatic systems at large and pose a hypothesis for the reason for the success of the α-form in nature.

## Introduction

Cyanobacteria are an ancient photoautotrophic lineage, whose origin precedes the great oxygenation event [1]. They have succeeded in colonizing habitats worldwide encompassing aquatic ocean and freshwater lake systems to extreme environments like hot springs through to terrestrial habitats including microbial mats from benthic ocean systems [2–6]. Via their possession of photosystems I and II, the latter capable of extracting electrons from water using light energy, ATP and reductant are generated that can be used to drive CO_2_ fixation through RuBisCO (ribulose 1,5-bisphosphate carboxylase/oxygenase). The resulting production of O_2_ has revealed a frailty of RuBisCO in that it cannot efficiently discriminate between the two substrates CO_2_ and O_2_. Thus, efficient CO_2_ fixation has required the development of CO_2_-concentrating mechanisms (CCMs) to increase the CO_2_ concentration around the active site of RuBisCO. For cyanobacteria, a major component of the CCM is a proteinaceous shell compartment, called the carboxysome, that surrounds RuBisCO [7, 8].

Whilst global cyanobacterial biomass is tiny compared to plant systems [9, 10], in marine systems cyanobacteria contribute around 25% of global marine primary production with oceanic productivity on a par with terrestrial ecosystems [11, 12]. Pico-sized cells of the genera *Prochlorococcus* and *Synechococcus* dominate such marine cyanobacterial production, being the two most abundant photosynthetic taxa on Earth [3, 11, 13]. As a result, these organisms have been widely studied in terms of their molecular ecology, physiology and genomics such that we now have a good mechanistic basis explaining their ecological success [14–16]. Both genera possess a Form IA RuBisCO and α-carboxysomes typifying these marine unicellular organisms as α-cyanobacteria [17, 18]. These are thought to be a product of horizontal gene transfer from α proteobacteria and exclusive to these taxa and marine environments [19]. In contrast, the common ancestor to all cyanobacteria presumably possessed a β-carboxysome and form IB RuBisCO since all other strains encompassing unicellular, filamentous and heterocystous lineages and including filamentous genera such as *Nostoc*, *Lyngbya*, *Anabaena*, *Planktothrix* or unicellular genera such as *Microcystis, Cyanothece, Synechocystis* and the *Synechococcus elongatus* clade are all β-cyanobacteria. The majority of these are freshwater, bloom-forming species.

Over recent years unicellular picocyanobacteria have been retrieved from freshwater environments which are phylogenetically much closer to their marine cluster 5 counterparts [20–23], that have likely escaped previous detection due to cultivation difficulties. Here, via sequencing the genomes of 58 novel freshwater isolates, all of which are phylogenetically related to cluster 5 picocyanobacteria from subclusters 5.2 and 5.3 [24], we demonstrate they all possess a form 1A RuBisCO and α-carboxysomes typical of α-cyanobacteria like their marine *Synechococcus* and *Prochlorococcus* counterparts. Using metagenomes from lakes across the globe, we show these cluster 5 freshwater picocyanobacteria are the dominant and most abundant phototrophs in pelagic areas of freshwater lakes/reservoirs worldwide. This work thus suggests these enigmatic cluster 5 members are the main pico-sized primary producers in freshwater systems, and that form 1A RuBisCO underpins CO_2_ fixation in this size fraction globally. Moreover, it eliminates salinity as an important environmental driver of the acquisition of α-carboxysomes and form 1A RuBisCO.

## Results

### A large set of new freshwater cluster 5 picocyanobacterial genomes

Following an isolation campaign of several years and subsequent purification of strains, we sequenced 58 new culture-derived freshwater picocyanobacterial isolates obtained from lakes and reservoirs across the world (Table S1). These spanned several continents including north Asia, central and western Europe, south-east Oceania and central and South America, and various trophic regimes such as the oligotrophic Lake Baikal (Russia), cold and glacial lakes (e.g., Lake Maggiore, Italy), meromictic lakes (Lake La Cruz and Lake El Tobar, Spain), temperate reservoirs (Tous, Loriguilla, Amadorio reservoirs, Spain) and tropical lakes (Lakes Atexcac or Alchichica, Mexico).

**Fig. 1:**
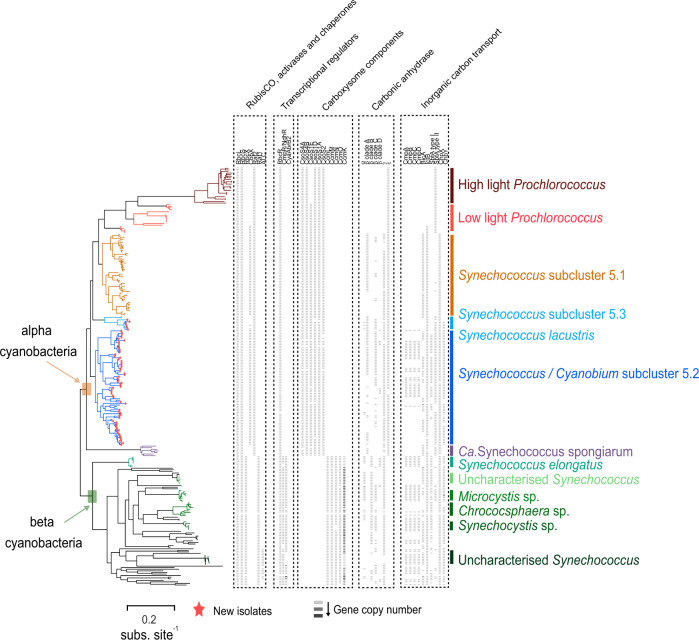
Phylogenomic tree of selected unicellular α and β cyanobacteria. New isolates used in this study are denoted by a red star. The tree is mid-point rooted. Gene presence/absence and copy number of RuBisCO, activases and chaperones, transcriptional regulators, carboxysome components, carbonic anhydrases and inorganic carbon transporters is indicated for each genome. The shade of gray signifies gene copy number, with genes in increased copy per genome given a darker shade.

Phylogenomics (Fig. 1) placed the majority of the isolates (a total of 52) inside SC 5.2, which comprises mainly freshwater and brackish/euryhaline/halotolerant strains [15, 20–23, 25]. Another six isolates phylogenetically comprised members of SC 5.3, recently proposed as a new genus *Ca*. Juxtasynechococcus [15], which includes marine RCC307/MINOS11 [15] and freshwater *S. lacustris* Tous [20] representatives. Perhaps unsurprisingly given their freshwater origin, none of the strains affiliated with members of subcluster 5.1 *Synechococcus* recently re-named *Ca*. Marinosynechococcus [15], *Prochlorococcus* or *Ca*. Synechococcus spongiarum. Amongst the new isolates, genome sizes ranged between ~2–4 Mbp, %GC content between 50–70% and a majority (35/58) were phycoerythrin-containing strains (Table S1). The remainder of the unicellular cyanobacterial genomes used in the phylogenomics analysis (Table S2), including *S. elongatus* and other *Synechococcus*-like genomes (mostly from the PCC clade), formed a phylogenetically distant and distinct clade compared to the herein presented new cluster 5 representatives (Fig. 1).

**Fig. 2:**
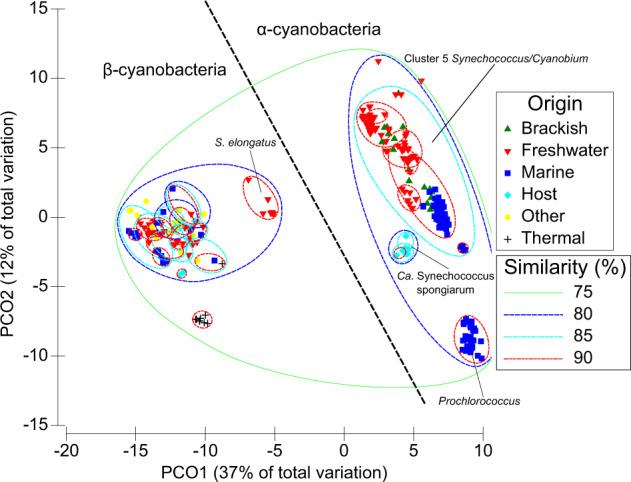
PCO plot obtained from a resemblance matrix based on SEED/KEGG gene presence/absence (Kulczynski index). The plot comprises 184 α-cyanobacteria and 88 β-cyanobacteria, all of them unicellular, labeled according to their habitat of origin.

The genomes were grouped using principle coordinates analysis based on KEGG/SEED gene presence/absence (Table S3). The first principle coordinate explains 37% of the variation, but does not separate these genomes by salinity preference (Fig. 2). Instead, cluster 5 picocyanobacteria grouped together at the right side of the ordination, slightly separated from *Ca*. Synechococcus spongiarum and *Prochlorococcus*, whilst to the left were other unicellular cyanobacteria comprising *S. elongatus*, other *Synechococcus*-like isolates as well as members of the genera *Microcystis*, *Synechocystis*, *Crocosphaera* and *Cyanothece*. To understand which genes drive the clear separation among the cyanobacteria, we compared the eigenvalues of each gene that correlated with the first principle coordinate. We found that virtually all of the high scoring genes (top-20 Eigenvalues) were involved in the formation of carboxysomes as well as RuBisCO components (Table S4). Beyond this, genomes tended to group by salinity or thermal tolerance. Thus, this analysis reinforces the classical separation of cyanobacteria into α- or β-cyanobacteria [7, 17, 18, 26], and led us to analyze in detail the composition and genomic context of carboxysome, RuBisCO and CCM components in these newly sequenced freshwater isolates as well as their marine/brackish cluster 5 relatives compared to their most immediate but distantly related *Synechococcus*-like freshwater relatives.

### The new freshwater cluster 5 picocyanobacterial isolates are all α-cyanobacteria possessing form IA RuBisCO and α-carboxysomes

**Fig. 3:**
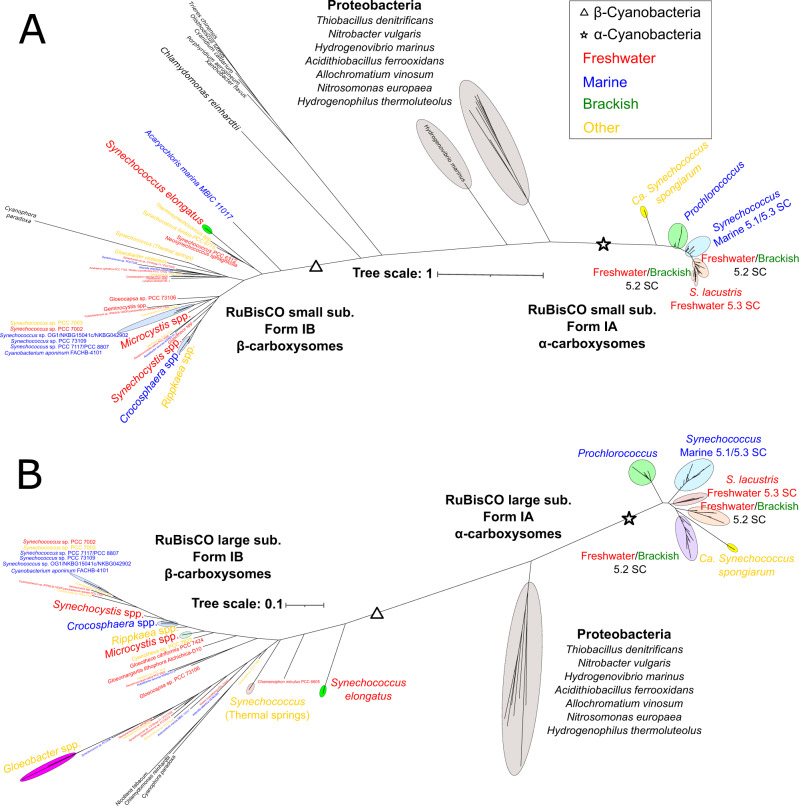
Large and small subunit RuBisCO phylogenies of the new freshwater genomes. **A** Unrooted RuBisCO small subunit phylogeny comprising unicellular/planktonic cyanobacterial strains and proteobacterial isolates. **B** Unrooted RuBisCO large subunit phylogeny comprising unicellular/planktonic cyanobacterial strains and proteobacterial isolates. Each isolate/group is color coded according to its habitat origin.

The phylogenomics (Fig. 1) and PCO analysis (Fig. 2) led us to establish the RuBisCO type present in these new freshwater cluster 5 picocyanobacteria. We compared 183 α-cyanobacteria comprising 17 brackish, 69 freshwater and 47 marine cluster 5 culture-derived picocyanobacteria, 42 *Prochlorococcus* isolates and 7 *Ca*. Synechococcus spongiarum MAGs, and a total of 83 unicellular β-cyanobacteria. Phylogenetic analysis using either the small or large subunit of RuBisCO (Fig. 3A, B) clearly showed the new isolates all possessed a proteobacterial-like form 1 A RuBisCO. Moreover, most of the new genomes (with the exception of some subcluster 5.3 strains) contained the RubisCO activase typical of most α-cyanobacteria, CbbX, whereas β-cyanobacteria possess the non-homologous RbcX type activase (Fig. 1). Similarly, all new genomes contained the pterin-dehydratase-like RuBisCO assembly factor, Raf2, but lacked the RuBisCO accumulation factor, Raf1, typical of β-cyanobacteria (Fig. 1 and Table S5). These non-homologous proteins play important but not fully characterized roles in assembling functional form 1A and 1B RuBisCO, respectively [27, 28].

**Fig. 4:**
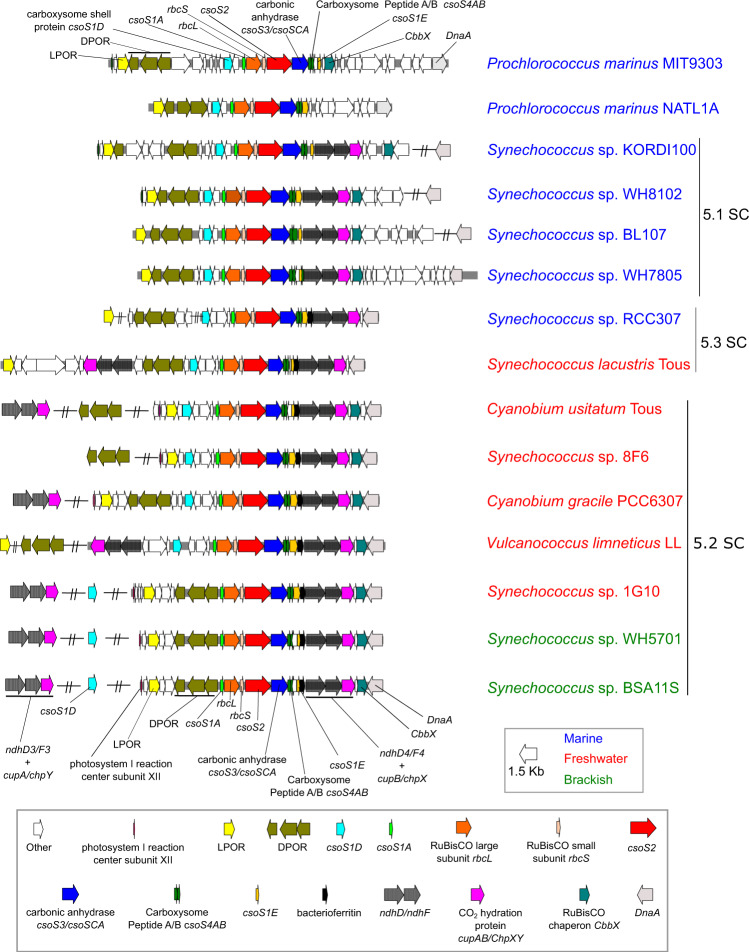
The genomic context of the α-carboxysome operon in cluster 5 picocyanobacteria included examples of the new freshwater genomes analyzed here. The habitat origin of each picocyanobacteria is color-coded accordingly. Breaks between genes display a separation in the genome or contig. Arrows are scaled (1.5 kb) and are color-coded according to the different genes they encode.

The new freshwater genomes also possessed the main components of α-carboxysomes including the carboxysome major shell protein CsoS1, the carboxysome assembly protein CsoS2, and shell vertex proteins CsoS4A and Cso4B (Fig. 1 and Table S5), comparable to what has been found in their marine SC 5.1 counterparts [29]. We next compared the structure of the carboxysome operon from the new freshwater genomes with examples of the same genomic region from *Prochlorococcus*, marine SC 5.1 *Synechococcus* and other brackish/freshwater *Synechococcus/Cyanobium* from SCs 5.2 and 5.3 (Fig. 4). Irrespective of their habitat of origin, all the new organisms showed a gene composition and genomic context consistent with them being α-cyanobacteria. The carboxysome shell proteins were clustered in the genome, all in the proximity of RuBisCO and the carboxysome associated ε-family carbonic anhydrase. Conversely, β-cyanobacteria showed a drastically different carboxysome operon structure. The genes encoding RuBisCO are rarely in the same context as those encoding the major shell components, CcmK1/2/3/4, CcmP, CcmL, CcmM, CcmN, CcmO (Fig. S1), unlike α-cyanobacteria. Instead, large (RbcL) and small (RbcS) RuBisCO subunits were clustered with the RuBisCO activase RbcX, whilst carbonic anhydrase was encoded disparately in the β genomes (Fig. S1).

### Freshwater α-cyanobacteria possess carbonic anhydrases previously associated with β-cyanobacteria

Carbonic anhydrases perform the interconversion between HCO_3_^−^ and CO_2_. They are therefore essential for increasing the local CO_2_ concentration in the carboxysome interior [30]. There are seven non-homologous families of carbonic anhydrase in nature, of which four are encoded by cyanobacteria: α, β, γ and ε (a.k.a ζ) [31]. β carbonic anhydrases can be further split into four phylogenetically distinct subfamilies (clades A-D, Fig. S2), which are all present in the cyanobacterial genomes analyzed here. Previously, α and β cyanobacteria displayed a clear distinction in carbonic anhydrase families [18]. The α-cyanobacterial clusters 5.1, 5.2, 5.3 and *Prochlorococcus* lacked α, β-B and β-D carbonic anhydrases, whereas β-A and β-C were sporadically distributed across cluster 5.1, 5.2 and 5.3, but absent from *Prochlorococcus* [18]. Instead, *Prochlorococcus*, and indeed all α-cyanobacteria possess a distinct family, ε, that is associated with the α-carboxysome and completely absent from β-cyanobacteria [32, 33]. This family, encoded by *csoSCA* or *csoS3* [34], is also found in alpha proteobacterial carboxysome operons, from whom α-cyanobacteria acquired it. In comparison, β-cyanobacteria were characterized by sporadic distribution of α, β-B, β-D and γ carbonic anhydrases [18].

**Fig. 5:**
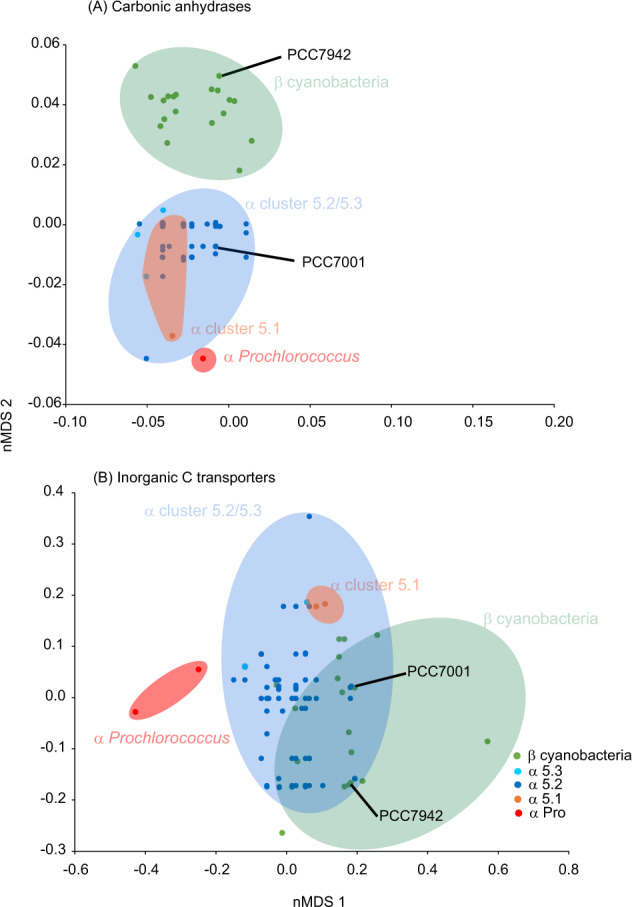
nMDS analysis of isolate genomes based on presence/absence of **A** carbonic anhydrases and **B** inorganic carbon transporters. Strains used in Whitehead et al. [18] are marked in the figure and discussed in the text.

Our new freshwater genomes contrast this previous division between α and β-cyanobacteria in carbonic anhydrase content. To support this, we produced individual phylogenies for each carbonic anhydrase type (Figs. S2–S4). The genomes from subclusters 5.2 and 5.3 sporadically contain α and β-D in addition to those previously identified in α-cyanobacteria (Fig. 1 and Table S5). Indeed, when performing non-metric multidimensional scaling analysis solely on carbonic anhydrase gene content, those genomes corresponding to cluster 5.2 and 5.3 form an intermediary between marine α cluster 5.1 and β-cyanobacteria (Fig. 5A). The phylogenies of both α and β-D carbonic anhydrases (Figs. S2 and S3), show orthologues that belong to α cyanobacteria cluster closely with β cyanobacteria of the genus *Synechococcus*, suggesting potential horizontal gene transfer from this group. Thus, for carbonic anhydrases, transfer from β cyanobacteria sharing the same freshwater environments may be common. For all other carbonic anhydrases, where both α and β cyanobacteria have a copy (β-C and γ), the phylogenies are completely congruent with the core (Figs. S3 and S4), and therefore strains that lack either may have lost these independently since the divergence of β and α cyanobacteria. Confirming previous work [17, 18] β-B are only found in β cyanobacteria, whereas β-A and ε are restricted to α cyanobacteria (Table S5) and thus it is impossible to determine evolutionary events that have led to this distribution.

### Inorganic C transporters

Experimentally determined cyanobacterial bicarbonate transporters comprise five systems that have largely been established mostly using the freshwater β-cyanobacterial model organisms *Synechocystis* sp. PCC6803 and *Synechococcus elongatus* PCC7942. These include: (1) the high-affinity bicarbonate transporter BCT1/CmpABCD, herein referred to as Cmp [35, 36]; (2) a medium to low affinity sodium dependent bicarbonate transporter of the SulP/SLC26 anion transporter family, called BicA [37–39]; (3) a member of the O-antigen ligase superfamily IctB [40]; (4) a proposed high-affinity sodium/bicarbonate symporter from the TC.2.A.83 sodium symporter family, SbtA [41–43], which can be split into two subfamilies SbtA1 and SbtA2 (Fig. S5); (5) two NADPH dehydrogenase (NDH-1) complexes that are involved in the uptake and recycling of CO_2_ by contributing to the accumulation of intracellular bicarbonate [44, 45]. NDH-I_3_ ChpY/CupA is a low CO_2_-inducible high-affinity CO_2_ acquisition system whilst NDH-I_4_ ChpX/CupB is involved in constitutive low affinity CO_2_ uptake [45]. Both systems are present in β-cyanobacteria. We note however, that for *ictB* no definitive biochemical studies demonstrate inorganic carbon transport and instead a role in polymer export has been suggested [46, 47].

Our analyses show that in addition to carbonic anhydrases, these new freshwater genomes are intermediaries between α and β-cyanobacteria in terms of these inorganic carbon transport systems (Fig. 5B). To support these observations, we also produced individual phylogenies for each inorganic C transport system (Figs. S5–S11). In particular, 29/76 members of subcluster 5.2 possess all subunits of the Cmp ABC-type transporter similar to the distribution in 50/83 β-cyanobacterial isolates (Figs. S6–S8 and Table S5). In contrast, this complex is completely absent from all marine α-cyanobacteria (subcluster 5.1 and *Prochlorococcus*) and freshwater subcluster 5.3. Similarly, the type I form of SbtA, SbtA1, is present in the majority of freshwater subcluster 5.2 and in β-cyanobacteria, but completely absent in subcluster 5.3 and marine α-cyanobacteria (Fig. S5 and Table S5). Further, ChpY follows a pattern similar to SbtA1, being present in β-cyanobacteria and freshwater α subcluster 5.2/5.3, but absent in all marine α subcluster 5.3, 5.1 and *Prochlorococcus* (Fig. S9 and Table S5). In contrast, whilst not present in every isolate, BicA (Fig. S10) and IctB (Fig. S11) are distributed throughout all β and α-cyanobacterial groups, but absent in *Prochlorococcus* (Table S5). This contrasts with SbtA2, which is present in members of every group, albeit in only two isolates of marine subcluster 5.1.

The protein phylogenies for CmpABCD (Figs. S6–S8), show freshwater α-cyanobacteria appear to have acquired this from β *Synechococcus* in the same fashion as carbonic anhydrases (Figs. S2–S4). The same is also true for *bicA* and *chpXY*, which have both subsequently been passed to marine subcluster 5.1 (Figs. S9 and S10). This contrasts the topologies for *ictB* (Fig. S11) and both forms of *sbtA* (Fig. S5), whose phylogenies are completely congruent with the core, suggesting these genes were present in the shared ancestor of α and β cyanobacteria and since lost in individual strains.

Thus, despite clearly being α-cyanobacteria (i.e., they possess an α form RuBisCO and carboxysome), our new isolates show greater similarity to β-cyanobacteria in both carbonic anhydrase and inorganic transporter systems (Figs. 5 and S12) and in some cases, horizontal gene transfer directly from β cyanobacteria explains this similarity.

### Cluster 5 α-picocyanobacteria globally dominate freshwater lakes

Given that all our new freshwater isolates are α-cyanobacteria, we sought to determine their global abundance and distribution in freshwater environments compared to their β-cyanobacterial relatives. Many previous studies have highlighted the global numerical dominance of the α-cyanobacterial genera *Synechococcus* and *Prochlorococcus* in marine systems [1, 13, 36], but work in freshwater systems has generally been lacking. However, a few studies have detected freshwater cluster 5 picocyanobacteria by FISH [25], 16S rRNA gene analysis [48, 49] and counting by epifluorescence microscopy or flow cytometry [4, 50, 51] in lakes all over the world.

**Fig. 6:**
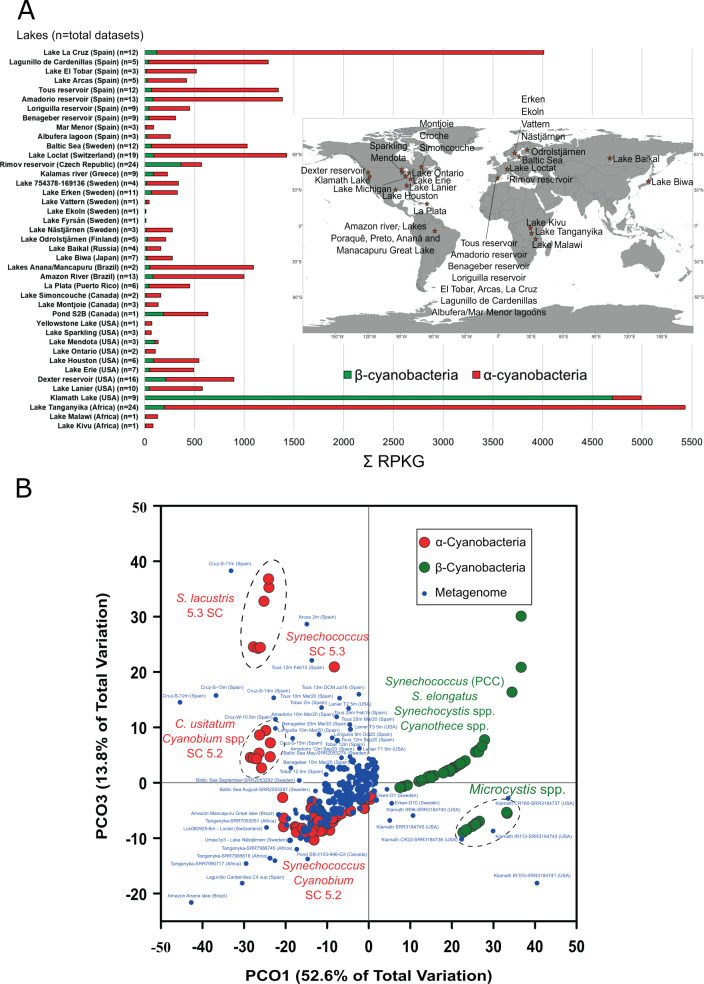
Global abundance of freshwater α and β-cyanobacteria in various lakes and reservoirs from across the globe. A total of 284 metagenomics datasets from lakes/reservoirs of varying trophic status and locations all over the world were used to assess abundance via read recruitment (RPKGs). **A** Map showing the locations of the metagenomics datasets (indicated by red stars) from which RPKG values were obtained. For each lake, we represent the number of used datasets with an *n*. The bar plot shows the total sum of RPKG values of 69 cluster 5 α-cyanobacterial isolates and 41 β-cyanobacterial strains. **B** PCO plot showing the different distribution of each isolate in all lakes, assessed as individual RPKG values for each strain.

Here, we used metagenomic recruitment analyses to detect both unicellular freshwater cluster 5 α and β-cyanobacteria in publicly available (SRA-NCBI) freshwater pelagic metagenomes, as well as 70 new metagenomes presented here (Supplementary Dataset 1). These metagenomes span fjords, bogs, lakes and reservoirs from various depths in the epi- and hypolimnion, include the deep chlorophyll maximum (DCM), and span a broad trophic status from ultra-oligotrophic to eutrophic. Geographically, they are derived from five continents (Fig. 6A). We used a range of cultured unicellular β-cyanobacteria and existing α-cyanobacteria (including those presented here), that represents the diversity of each group (see Fig. 6 and Supplementary Dataset 1), to map reads from metagenomes against. We express the relative abundance of each genome in each metagenome as reads per kilobase of genome per gigabase of metagenome (RPKG) (see “Methods” for further details). In 93% (263/284) of metagenomes, α-cyanobacteria had greater RPKG values than β-cyanobacteria. In each metagenome, the median RPKG values for α-cyanobacteria were seven times greater than β-cyanobacteria (Wilcoxon signed rank test, *z*_284_ = −9.9073, *p* < 0.001).

Among the globally dominant α-cyanobacteria, noteworthy were two cluster 5 freshwater groups that were detected in the majority of the assessed freshwater metagenomes all over the globe (Fig. S13 and Supplementary Dataset 1). These two groups comprise a cluster of *Cyanobium* spp. from SC 5.2 (including *C. usitatum* as the type species) and another group from SC 5.3 comprising mainly *S. lacustris* species, which are well-known cosmopolitan and widespread species [20]. In the few exceptions (21/287) where β-cyanobacteria had greater RPKG values than α, the majority of reads mapped to genomes of *Microcystis* spp. (β-cyanobacteria). These derived from Lakes Vattern, Ekoln and Fyrsan (Sweden) or Lakes Mendota and Klamath (USA). We suspect these lakes were being subjected to *Microcystis* bloom events of members of this potentially toxic genus, since no other cluster 5 picocyanobacterial members were detected at these locations. Apart from these ephemeral *Microcystis* blooms, that naturally occur in eutrophic lakes under certain conditions [52, 53], no other unicellular and filamentous β-cyanobacterial species were significantly detected in the 41 different systems with ca. 284 metagenomes analyzed (Supplementary Dataset 1 and Fig. 6B). This leads us to conclude that unicellular α-cyanobacteria from cluster 5 dominate freshwater aquatic ecosystems worldwide with the exception of some eutrophic lakes where sporadic bloom-forming β-cyanobacteria dominate.

## Discussion

Cyanobacteria are key primary producers in aquatic habitats worldwide [3, 4, 11, 51]. Unicellular forms numerically dominate such environments with the accepted general rationale being that α-cyanobacteria occupy marine systems and β-cyanobacteria freshwater environments [18, 26, 29]. This work challenges such a paradigm by demonstrating that in fact α-cyanobacteria dominate aquatic habitats (both marine and freshwater) globally. Why, therefore, do two forms of carbon fixation machinery exist in the cyanobacteria, and why does the recently acquired α form dominate aquatic systems? Previous studies comparing the biochemistry of single representatives of α and β-cyanobacterial RuBisCOs, have shown identical catalytic rates between the two forms of the enzyme [54]. Meanwhile, although α-carboxysomes are generally physically smaller than their β counterparts, their increased copy number per cell leads to identical functioning [18]. One major genomic difference between α and β cyanobacteria analyzed here is genome size and intergenic spacer lengths (Fig. S14). α-cyanobacteria (regardless of their origin) have smaller genome sizes and smaller median intergenic spacers compared with β (Fig. S14), indicative of a K-strategist lifestyle (oligotrophs/persisters), compared with r-strategists (copiotrophs/bloomers). However, it is not clear how these two life-history traits would select for the two CCM machinery types, given their functional similarities [18]. Here, we show salinity is unlikely the driving force leading to the diversification of α-cyanobacteria in today’s aquatic systems, given that the α form dominates large water masses across the salinity divide.

**Fig. 7:**
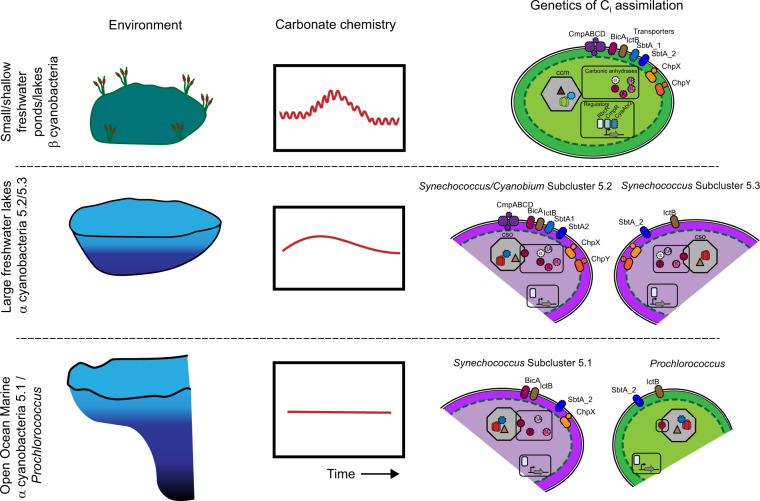
Proposed model for the evolution of α and β cyanobacteria in aquatic environments. Each environment and associated fluctuation in carbonate chemistry is shown on the left. On the right, the presence of RuBisCO, carboxysome, transcriptional regulators, carbonic anhydrases and C_i_ transport systems are shown. In each case, a solid line around each protein denotes its presence in all taxa within the group, whilst dashed symbolizes presence in at least one member of the group.

We thus explored other differences in environments dominated by α and β-cyanobacteria. Pertinent to inorganic carbon assimilation by the Calvin cycle, we considered differences in carbonate chemistry and oxygen concentration between shallow, small lakes, puddles and ponds (β dominated) and large lakes and oceans (α dominated) (Fig. 7). Large freshwater lakes form strong epilimnetic layers during the summer and may therefore be seasonally more geochemically similar to upper ocean ecosystems. Indeed, a recent database of mean pH values from 12,934 freshwater lakes worldwide determined an average value of 7.99 [55], confirming the relevance of such moderate alkalinity globally. Such conditions have been observed in the largest and deepest freshwater lake in the world, Lake Baikal, typically showing a profile from neutrality to slightly alkaline [56], alkaline epilimnions in meromictic Spanish lakes such as La Cruz [57, 58] or El Tobar [59] and small Spanish inland lakes [60], Mexican crater lakes such as Atexcac and Alchichica [61] or photic layer and DCMs from Spanish reservoirs [20, 25, 62–64], from which several of our isolates were obtained. This tendency to alkalinity mirrors the situation in the ocean (pH 8.2 ± 0.3 in spite of growing acidification [65]). The strong influence of pH in dictating the energetics of CCM systems [66] might well explain why these small phototrophs have developed their CCMs to cope and perform optimally under neutral to alkaline conditions where bicarbonate is the most abundant inorganic carbon form, leading to their colonization of virtually all aquatic habitats across the globe (Fig. 7). In contrast, small, shallow lakes and ponds that do not form pelagic strata show rapid daily and seasonal fluctuation in carbonate chemistry and oxygen (Fig. 7) [67, 68]. Indeed, pH levels in small ponds can vary over two orders of magnitude in a single day [68], resulting in rapidly fluctuating proportions of CO_2_, HCO_3_^−^ and CO_3_^2−^ and also major shifts in population density with frequent crashes followed by periods of high growth rates (blooms). Similarly, episodic nutrient influxes from anthropogenic activities lead to transient eutrophication, which perturbs carbonate and oxygen chemistry [69]. Accordingly, β- cyanobacteria harbor an increased diversity of inorganic carbon transport mechanisms, carbonic anhydrases and inorganic carbon responsive transcriptional regulators (Figs. 1, 5, 7 and S12 and Table S5). Our freshwater α genomes form an intermediary between freshwater β and marine α-cyanobacteria in terms of both carbonic anhydrase content (Fig. 5A) and inorganic carbon transport (Fig. 5B). This is despite freshwater and marine α-cyanobacteria sharing a common ancestor (Fig. 1), whilst β-cyanobacteria are thought to pre-date α [70], with the α form originating ca. 1 bya. Reconstructions of marine carbonate chemistry do not extend back this far [71, 72], but due to their size, it is likely that marine environments have never fluctuated rapidly in carbonate chemistry. Here we describe a scenario, where α-cyanobacteria have come to dominate temporally stable large lakes and oceans, whereby this transition has been accompanied by a shift in the diversity of inorganic carbon transport systems, carbonic anhydrases and ultimately the carboxysome and RuBisCO itself (Fig. 7). Indeed, supporting this idea, all α-cyanobacteria lack the C_i_ transcriptional regulators CmpR and CyaAbr2 (Fig. 1). We posit that the α machinery represents a specialized solution to stable carbonate and oxygen chemistry, whereas the β machinery is a “jack of all trades”, capable of operating efficiently in a rapidly fluctuating C_i_ and O_2_ environment. Measurements of carboxysome performance are scarce, yet, Whitehead et al. [18] compared the response of a β cyanobacterium (*Synechococcus* sp. PCC7942) with a salt-adapted (brackish) α-cyanobacterium (*Cyanobium* sp. PCC7001) to changes in *p*CO_2_. They show the α cyanobacterium seems to lack the ability to control many facets of cellular physiology in response to differing *p*CO_2_. For example, on a per cell basis the maximum activity (*V*_max_) of RuBisCO was unchanged in the α, whereas the *V*_max_ in the β was increased 1.64 fold. Similarly, the internal C_i_ pool is unchanged in the β in both high and low CO_2_ grown cells, whereas a dramatic increase in Ci is observed in the α cyanobacterium when grown under low CO_2_. Nevertheless, the authors conclude that carboxysome and RuBisCO functioning per se were remarkably similar [18]. We note however, that *Cyanobium* sp. PCC7001 (brackish/halotolerant) is not particularly representative of freshwater α-cyanobacteria in terms of C_i_ uptake mechanisms (Fig. 5B), and this study is restricted to single members of each group, whilst later work has reinforced the absence of induction of the carboxysome in low CO_2_ in several α-cyanobacteria [71]. Ultimately, further work that compares the performance of α and β-cyanobacteria in response to carbonate chemistry more broadly is required to test our hypothesis.

Understanding why these two forms exist has importance for not only understanding the Earth’s early carbonate chemistry, when these systems evolved, but also they may be important for predicting the biosphere’s response to projected increases in *p*CO_2_ and the resulting decrease in pH many of our oceans face.

## Materials and methods

### Isolation of new freshwater picocyanobacteria

The novel freshwater strains described here were obtained across a 5 year period using previously described isolation approaches [20–23]. All isolates were ultimately grown in either normal or two-fold diluted BG-11 medium [73]. Briefly, to obtain them, we applied techniques such as dilution to extinction, filtration and flow cytometric single-cell sorting (InFlux V-GS flow cytometer, Becton Dickinson Inc.). However, in all cultures picocyanobacteria represented >75% of all cells as monitored by flow cytometry, microscopy and recovered genomic data [23]. All isolates are available from the MEG-Verbania [23] and University of Valencia cyanobacterial culture collections.

### DNA extraction and sequencing, read assembly, contig annotation and obtaining of draft genomes

DNA from the newly described freshwater strains was extracted using two different methods: either using the EZNA soil DNA extraction kit (Omega Bio-Tek) or a CTAB-lysis buffer followed by phenol-chloroform-isoamyl alcohol extraction approach [74], the latter generally providing higher DNA recovery.

Genomic DNA was sequenced using a NovaSeq (Illumina, USA) PE150/MiSeq (Illumina, USA) PE250 and Illumina DNA library preparation technology (Novogene, UK/Hong Kong). Approximately 1 Gb sequence data was obtained for each isolate. Sequence data was individually trimmed with Trimmomatic v0.39 [75], assembled with SPAdes v3.13.1 [76] following --careful, --only-assembler, -k 57,67,77,87,97,107,117,127, -t 48, -m 250 parameters. Assembled contigs were manually inspected to remove heterotrophic bacterial sequences and to uniquely bin the contigs belonging to each cyanobacterial strain. To do so, firstly ORF prediction was assessed using Prodigal v2.6.3 [77], whilst the functional annotation and taxonomy of each CDS and contig was assessed with BLAST (nr database) using Diamond v2.0.6.144 [78]. Proteins were annotated using the latest NCBI nr, KEGG [79], SEED [80], COG [81] and TIGRFAMs [82] databases to provide the most robust nomenclature and taxonomy. With this information we manually inspected all contigs and separated cyanobacteria from heterotrophic bacteria when >50% of CDS hits belonged to the cyanobacterial phylum. Then, a further step of Metabat2 v2.14 [83] was applied to bin cyanobacterial contigs into draft genomes. checkM v1.1.3 [84] and GTDB [85] were also used to estimate the completeness and phylogenetic placement of each genome.

### Phylogenomics of unicellular cyanobacteria

Phylogenomics used a 370 protein concatenated tree obtained via the PhyloPhlAn3 tool [86] using the following parameters: -t a --diversity high --accurate -f configs/supermatrix_aa.cfg. This analysis exclusively used culture derived (either complete or draft genomes) marine (48 genomes), brackish (17 genomes) and freshwater (69 genomes) picocyanobacteria from subclusters 5.1, 5.2 and 5.3. All marine/halotolerant *Synechococcus* isolates were derived from the Cyanorak database [87] together with 42 *Prochlorococcus* genomes from the same database. 8 *Ca*. Synechococcus spongiarum MAGs [88–90] and 88 different unicellular β-cyanobacteria were used including *S. elongatus* [89], *Gloeomargarita lithophora*, *Gloeobacter kilaueensis/violaceus*, *Gloeocapsa* spp., *Microcystis* spp. *Synechocystis* spp., *Thermosynechococcus*, *Crocosphaera* spp., *Geminocystis* spp., *Acaryochloris* spp., *Cyanothece* spp., *Synechococcus*-like Yellowstone isolates and other unicellular strains from subsection I [91].

We also used the abovementioned isolates to perform a first search of individual genes/proteins presence/absence against the KEGG [79] and SEED [80] databases (Table S3). We used diamond v2.0.6.144 BLASTP/BLASTX searches with >75% query coverage and >30% sequence identity. A PCO was then obtained from a resemblance matrix based on SEED/KEGG gene presence/absence (Kulczynski index).

### Homology searching

For RuBisCO and carboxysome components, we used diamond blastp searches with known orthologues at >75% query coverage, >30% identity [78]. Sequences for inorganic carbon transporters and carbonic anhydrases are poorly conserved. Thus, to search for distant homologs between α and β taxa, conserved domains were searched for using RPSBLAST v2.13. Pre-computed PSSMs for each protein of interest were used. Candidate hits were subsequently used in phylogenetic analyses below to assign putative function. A presence/absence matrix containing all of these individual genes is shown in Table S5.

### RuBisCO, carbonic anhydrases and inorganic C transporter individual phylogenetic trees

Individual phylogenies of the different RuBisCO subunits, bicarbonate transporters and carbonic anhydrases were obtained by aligning individual proteins with MAFFT v7.490, using default parameters and 1000 iterations [92]. Alignments were manually inspected. Phylogenies were constructed in FastTree v2.1 [93], using the JTT + CAT model.

### Sampling and metagenomics sequencing

For the metagenomes newly presented in this study Spanish lakes and reservoirs were sampled in two different seasons (winter-mixed and summer-stratified periods) and for each lake/season representative samples corresponding to the epilimnion, hypolimnion and DCM (for the summer period) were obtained. This allowed us to monitor the abundance of α- and β-cyanobacteria at different times of the year. No blooms of β-cyanobacteria were detected in any of the Spanish lakes from which metagenomes were obtained. Further details of sampling metadata, including the depth and sample location are given in Supplementary Dataset 1. Pelagic water samples from the different Spanish lakes (Lakes La Cruz, Cardenillas, Arcas and El Tobar, and Tous, Loriguilla, Amadorio and Benageber reservoirs) were obtained through a 3-year sampling campaign. Briefly, 20 l water were sequentially filtered through 20, 5 and 0.22 µm pore size filters and DNA extracted with CTAB-lysis buffer followed by phenol-chloroform-isoamyl alcohol extraction [74]. We exclusively sequenced (NovaSeq (Illumina, USA) PE150, Novogene UK) the small plankton fraction that passed through the 5 µm pore size filter but which was retained on the 0.22 µm pore size filter. Approximately 15 Gb/output (ca. 100 million reads) were obtained for each metagenome.

### Metagenomics read recruitment analysis across freshwater lakes

We used a total of 284 metagenomes from 41 different lakes that reasonably cover the entire globe. The different metagenomics datasets we used, most of which comprise chronoseries of different seasons/depths (fine profiles), where we detected the significant presence (>2 RPKGs) of α/β-cyanobacteria were those coming from Spanish reservoirs, Mediterranean coastal lagoons, Lake Baikal, USA lakes and reservoirs, Canadian lakes, Lake Tanganyika, tropical Amazonian lakes and rivers, Lake Biwa, the Baltic Sea, North-European and central European lakes and rivers (see Supplementary Dataset 1). We assessed the global abundance of each unicellular freshwater cluster 5 α and β-cyanobacteria using metagenomics read recruitment, as previously described [20, 24]. Briefly, we mapped individual metagenomics reads from each freshwater lake/reservoir to each genome, exclusively validating the presence of hits using parameters of >95% sequence identity and >50 bp alignment length between the genome and metagenome read. These hits were counted as reads per Kb of genome per Gb of metagenome (RPKGs) (see Supplementary Dataset 1). We used a recruitment threshold of >2 RPKGs to determine the abundance of each α/β-cyanobacterial isolate.

To assess if differences in RPKGs between lakes were statistically significant we constructed a Bray-Curtis resemblance matrix based on the abundance RPKG values for each strain in each lake using the PRIMER6 tool [94]. Using the derived triangular matrix, we then performed a PCO plot where genomes were distributed accordingly and each lake correlation was also shown and plotted (Fig. 6B).

## Supplementary information

Supplementary Figure Legends

Figure S1

Figure S2

Figure S3

Figure S4

Figure S5

Figure S6

Figure S7

Figure S8

Figure S9

Figure S10

Figure S11

Figure S12

Figure S13

Figure S14

Supplementary Data 1

Table S1

Table S2

Table S3

Table S4

Table S5

## Data Availability

All data derived from this work are publicly available in NCBI-Genbank databases. Genomes of all the newly sequenced freshwater cluster 5 picocyanobacterial cultures have been deposited in the NCBI-Genbank database under Bioproject number PRJNA718564, biosample numbers SAMN18541576-SAMN18541633 and Genbank accession numbers JAGQAY000000000-JAGQDB000000000. Additionally, the newly presented 70 metagenomes from Spanish lakes and reservoirs have been deposited under Bioproject numbers PRJNA721863, PRJNA745587, PRJNA745573, PRJNA745574 and PRJNA639779.
