# Supplementary material for: α-cyanobacteria possessing form IA RuBisCO globally dominate aquatic habitats
Source: ISME J. 2022 Jul 18;16(10):2421–32. doi: 10.1038/s41396-022-01282-z (PMC9477826; doi:10.1038/s41396-022-01282-z)
Supplement: Supplementary file 1 — Supplementary Figure Legends [file 41396_2022_1282_MOESM1_ESM.docx]

**Supplementary Figure Legends:**

**Figure S1.** The genomic context of the β-carboxysome operon in various unicellular cyanobacterial model organisms. The habitat origin of each cyanobacteria is colour-coded accordingly. Breaks between genes display a separation in the genome or contig. Arrows are scaled (1.5 kb) and are colour-coded according to the different genes they encode.

**Figure S2.** Protein based beta carbonic anhydrase phylogeny including α- (red-coloured) and β-cyanobacterial (green-coloured) isolates studied in this work. Tree was rooted at the mid-point.

**Figure S3.** Protein based alpha carbonic anhydrase phylogeny including α- (red-coloured) and β-cyanobacterial (green-coloured) isolates studied in this work. Tree was rooted at the mid-point.

**Figure S4.** Protein based gamma carbonic anhydrase phylogeny including α- (red-coloured) and β-cyanobacterial (green-coloured) isolates studied in this work. Tree was rooted at the mid-point.

**Figure S5.** Protein based SbtA phylogeny including α- (red-coloured) and β-cyanobacterial (green-coloured) isolates studied in this work. Tree was rooted at the mid-point.

**Figure S6.** Protein based CmpA/NrtA phylogeny including α- (red-coloured) and β-cyanobacterial (green-coloured) isolates studied in this work. Tree was rooted at the mid-point.

**Figure S7.** Protein based CmpB/NrtB phylogeny including α- (red-coloured) and β-cyanobacterial (green-coloured) isolates studied in this work. Tree was rooted at the mid-point.

**Figure S8.** Protein based CmpCD/NrtCD phylogeny including α- (red-coloured) and β-cyanobacterial (green-coloured) isolates studied in this work. Tree was rooted at the mid-point.

**Figure S9.** Protein based ChpXY phylogeny including α- (red-coloured) and β-cyanobacterial (green-coloured) isolates studied in this work. Tree was rooted at the mid-point.

**Figure S10.** Protein based BicA phylogeny including α- (red-coloured) and β-cyanobacterial (green-coloured) isolates studied in this work. Tree was rooted at the mid-point.

**Figure S11.** Protein based IctB phylogeny including α- (red-coloured) and β-cyanobacterial (green-coloured) isolates studied in this work. Tree was rooted at the mid-point.

**Figure S12.** Revised generalized schematic of α and β-cyanobacteria based on the new genomic information from freshwater isolates obtained here. Each type of protein/CCM system is marked accordingly. Different groups are established according to their possession of specific inorganic carbon transporter components. +/- indicates that particular system was not present in all genomes analyzed.

**Fig. S13.** Global abundance of the main groups of freshwater α- and β-cyanobacteria in various lakes and reservoirs from across the globe. The locations from which metagenomic datasets were obtained are indicated with red stars. The bar plot shows average RPKG values of 67 culture-based cluster 5 α-cyanobacteria isolates, sub-divided into i) SC 5.3 (*S. lacustris* isolates), ii) SC 5.2 *Cyanobium* spp (with *C. usitatum* as the main representative isolate), iii) remaining SC 5.2 freshwater strains, and 41 β-cyanobacteria strains (*Microcystis* spp, *Synechocystis* spp., *S. elongatus*).

**Fig. S14.** Estimated genome sizes (Mb) versus median intergenic spacer length (bp) and %GC content between α and β cyanobacteria.
