# Supplementary figures and images for: α-cyanobacteria possessing form IA RuBisCO globally dominate aquatic habitats
Source: ISME J. 2022 Jul 18;16(10):2421–32. doi: 10.1038/s41396-022-01282-z (PMC9477826; doi:10.1038/s41396-022-01282-z)

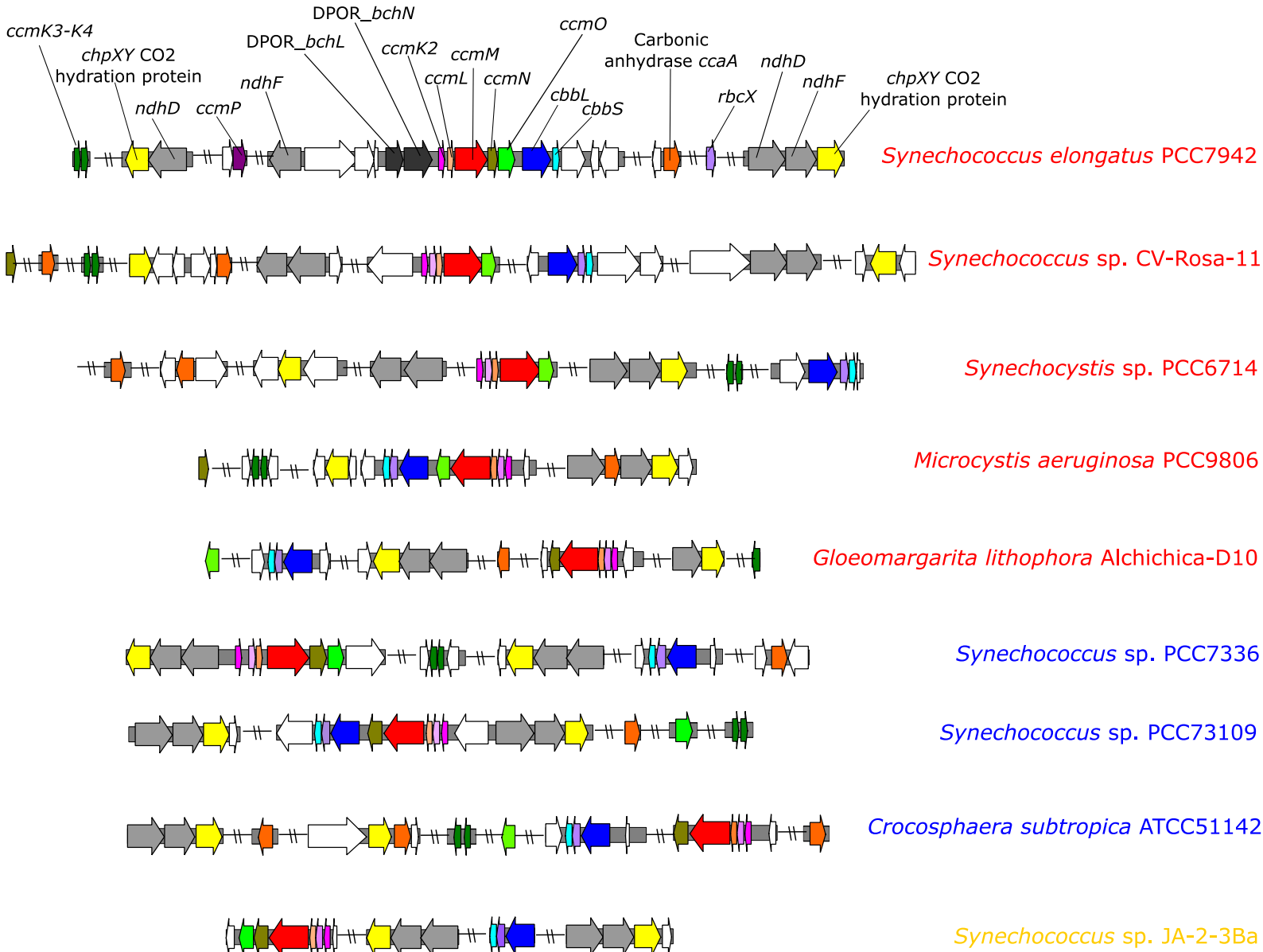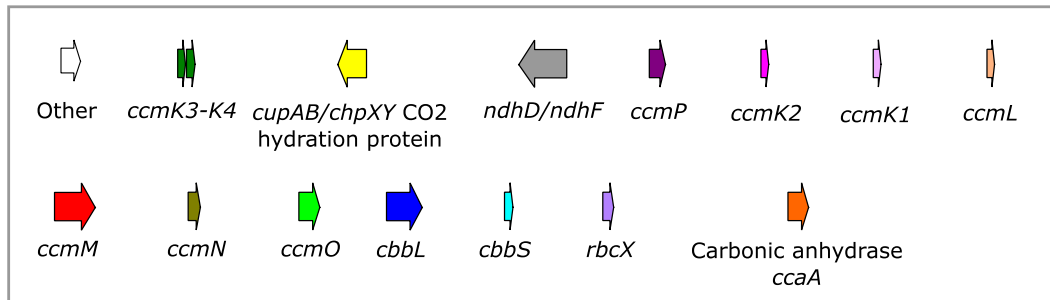

1.5 Kb

Freshwater  
Marine  
Thermal springs

Supplement: Supplementary file 2 — Figure S1 [file 41396_2022_1282_MOESM2_ESM.pdf]

Tree scale: 1

bootstrap

- 0
- 0.25
- 0.5
- 0.75
- 1

clade D

clade B

clade A

clade C

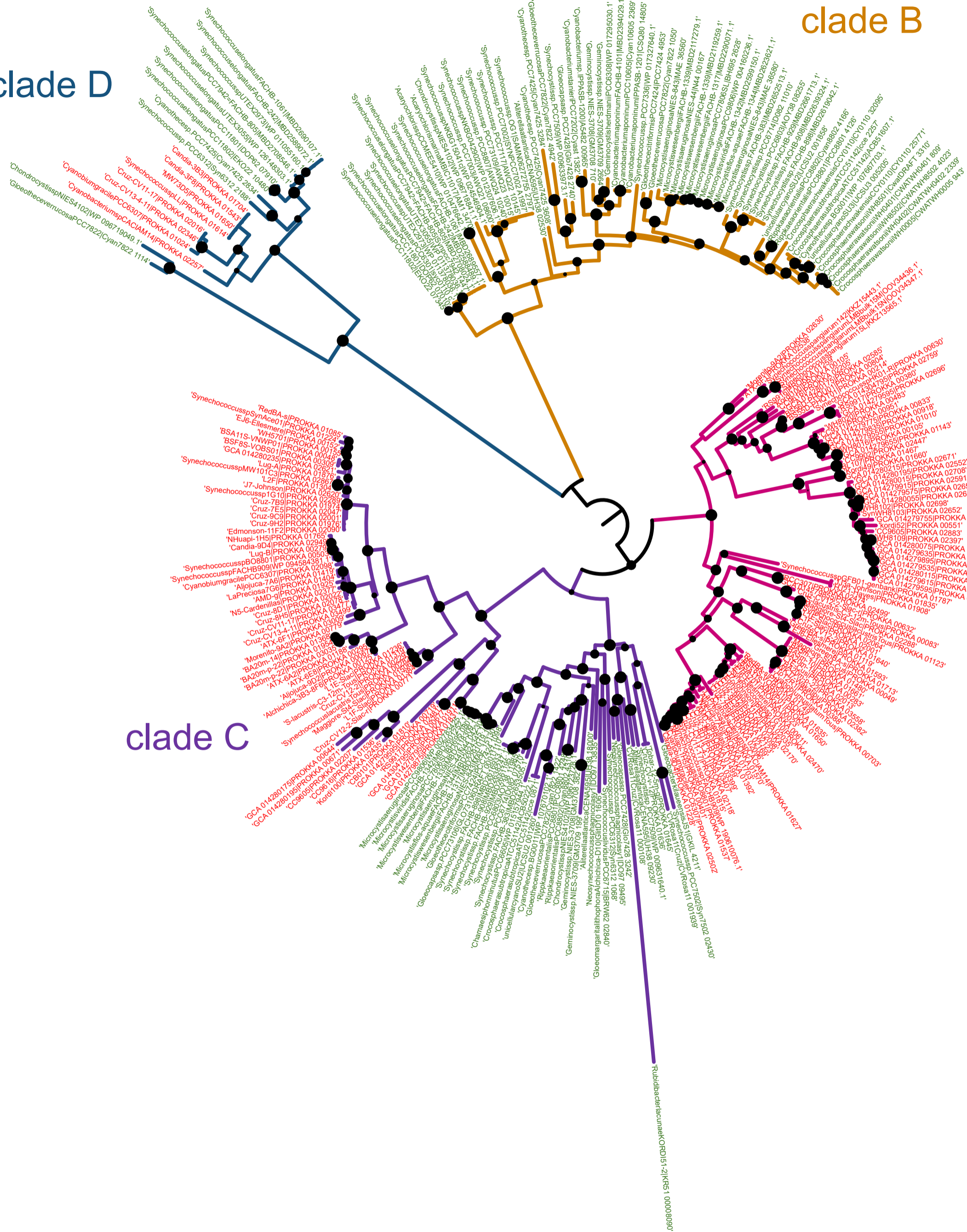

Supplement: Supplementary file 3 — Figure S2 [file 41396_2022_1282_MOESM3_ESM.pdf]

Tree scale: 1 

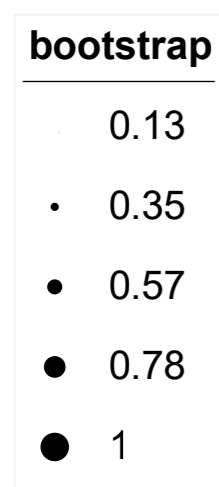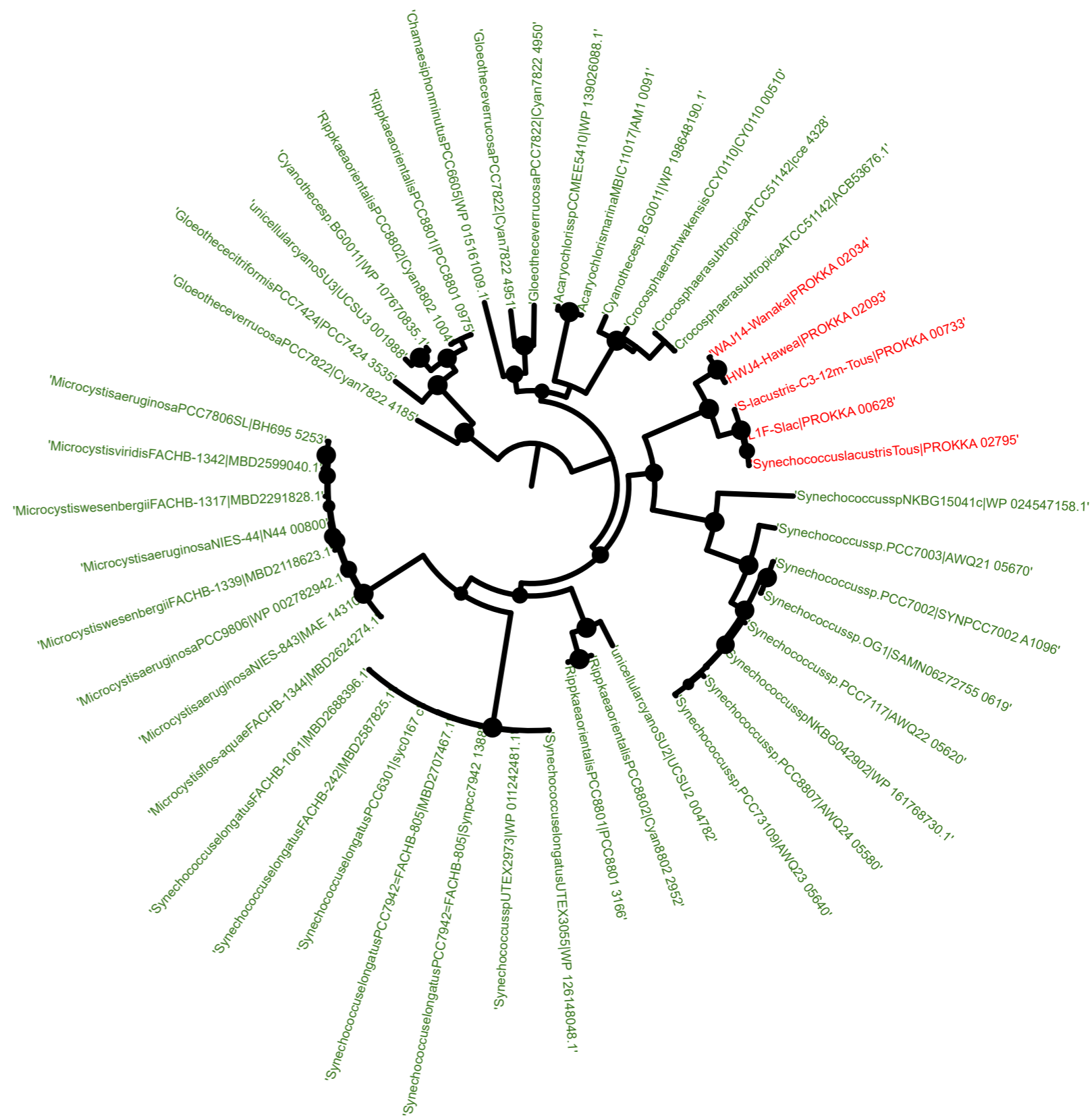

Supplement: Supplementary file 4 — Figure S3 [file 41396_2022_1282_MOESM4_ESM.pdf]

Tree scale: 1

bootstrap

- 0
- 0.25
- 0.5
- 0.75
- 1

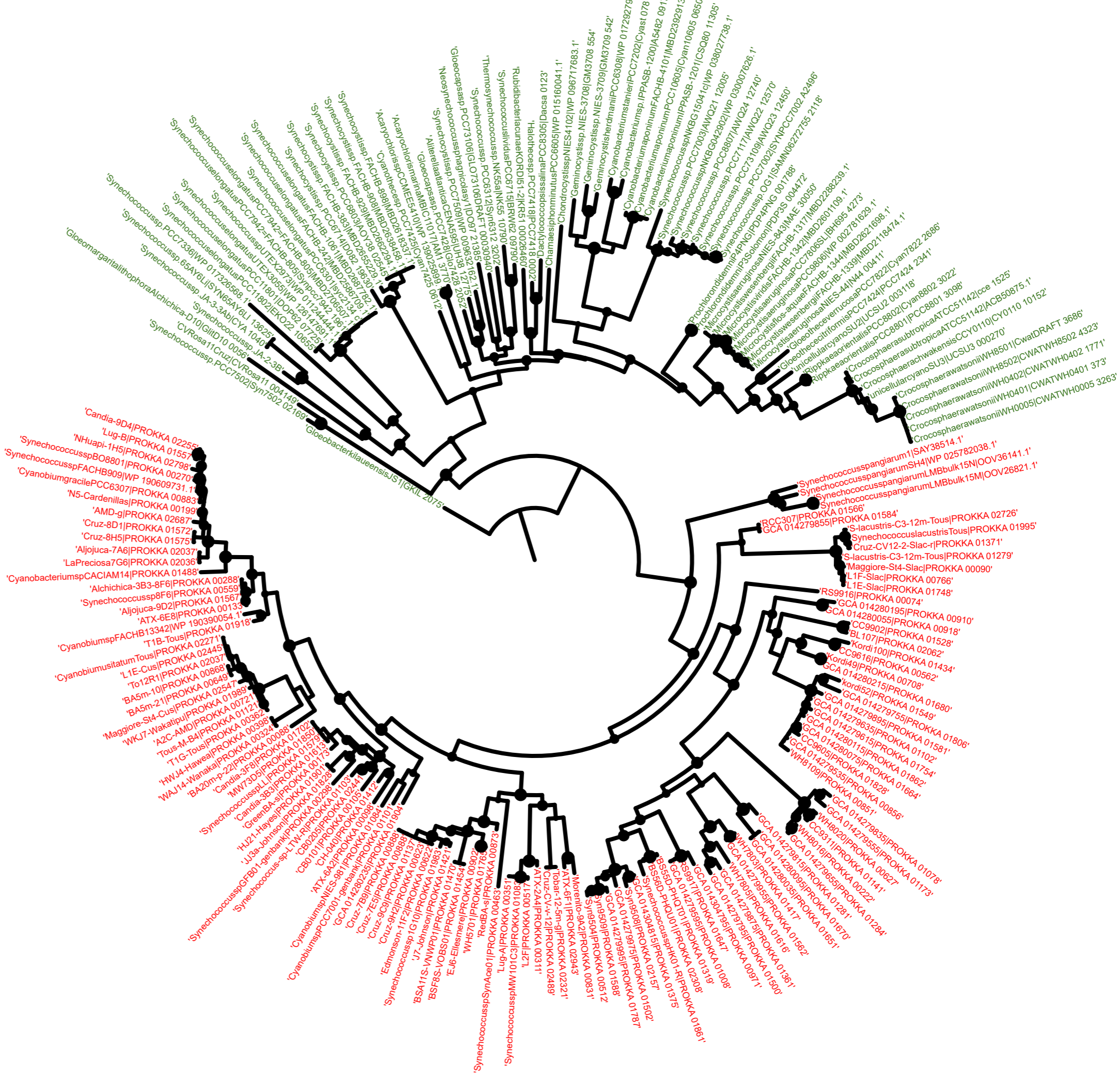

Supplement: Supplementary file 5 — Figure S4 [file 41396_2022_1282_MOESM5_ESM.pdf]

sbtA Type I

Tree scale: 1

bootstrap

0

• 0.25

• 0.5

• 0.75

• 1

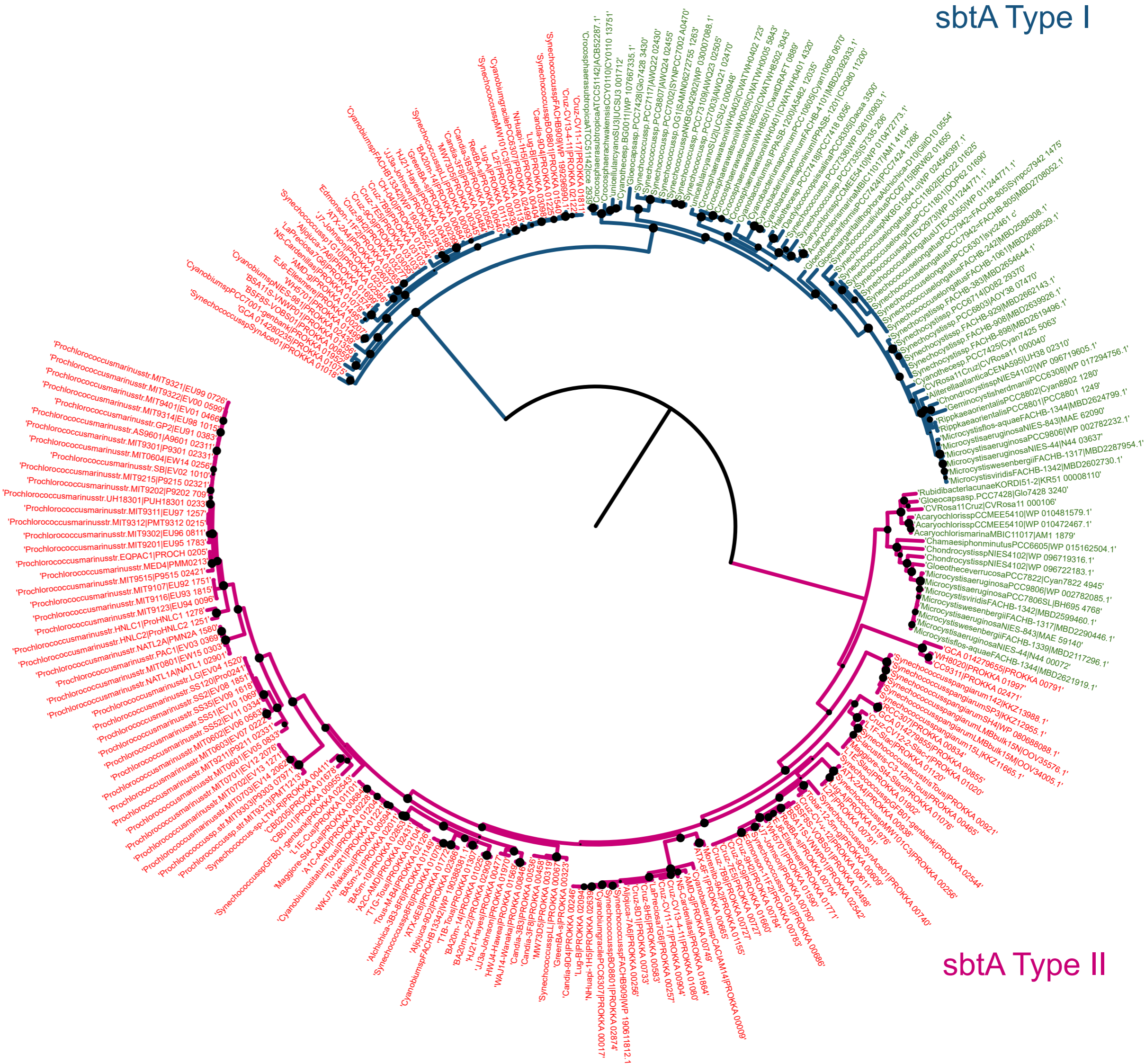

sbtA Type II

Supplement: Supplementary file 6 — Figure S5 [file 41396_2022_1282_MOESM6_ESM.pdf]

## cmpA

**Tree scale: 1**

## bootstrap

0

0.25

0.5

0.75

1

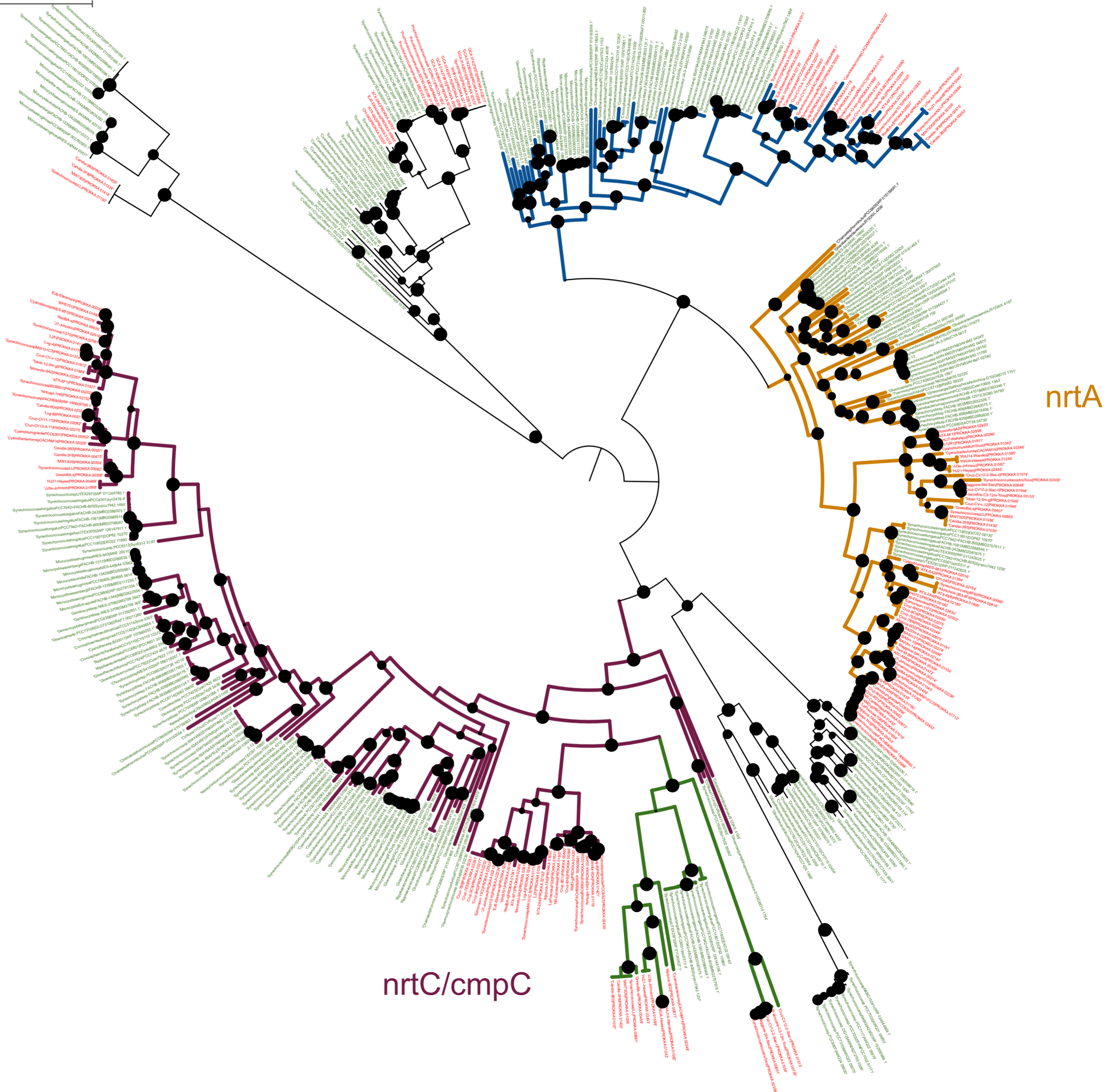

Supplement: Supplementary file 7 — Figure S6 [file 41396_2022_1282_MOESM7_ESM.pdf]

Tree scale: 1

bootstrap

- 0
- 0.25
- 0.5
- 0.75
- 1

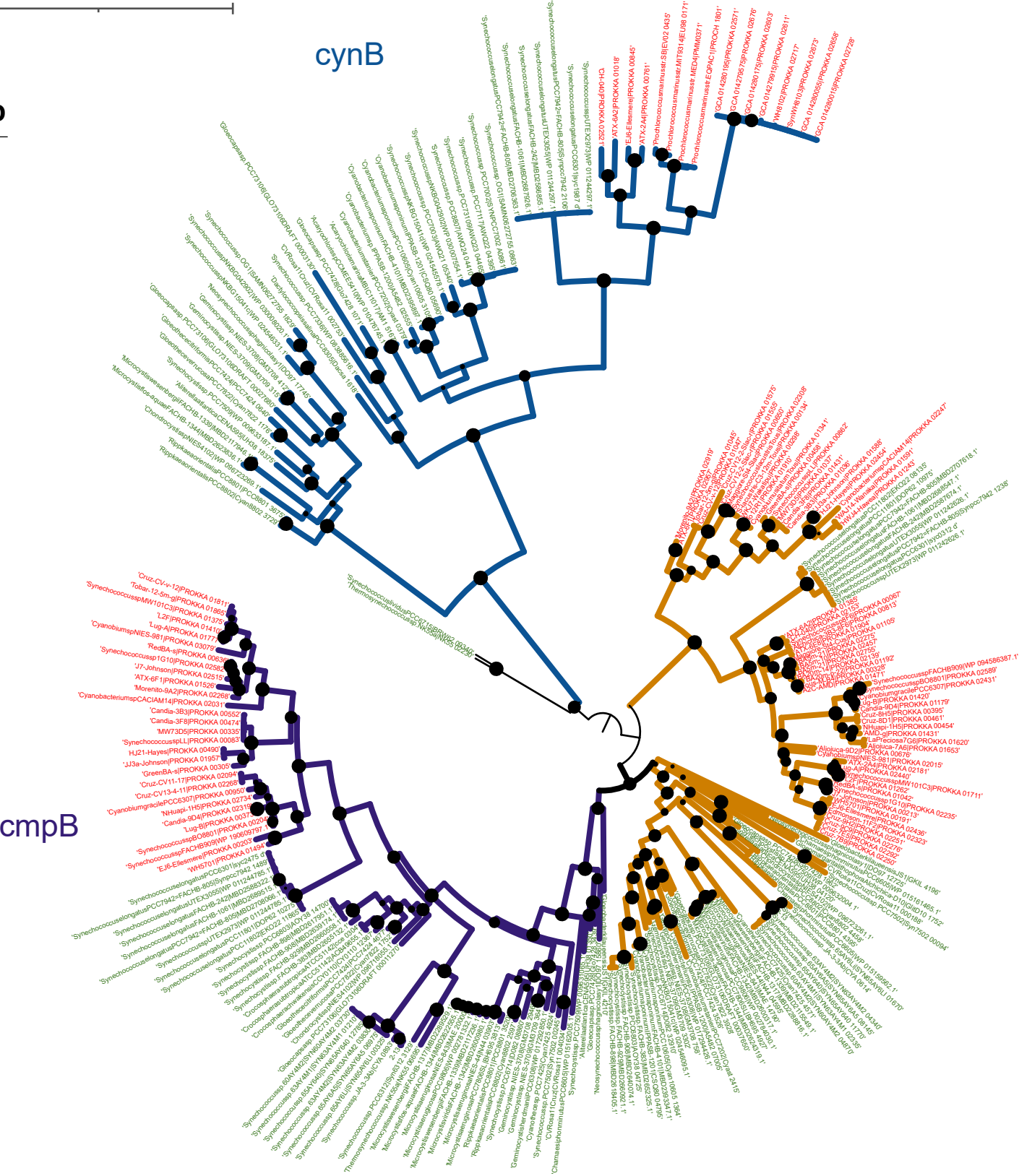

*cynB*

*cmpB*

*nrtB*

Supplement: Supplementary file 8 — Figure S7 [file 41396_2022_1282_MOESM8_ESM.pdf]

Tree scale: 1

bootstrap

- 0
- 0.25
- 0.5
- 0.75
- 1

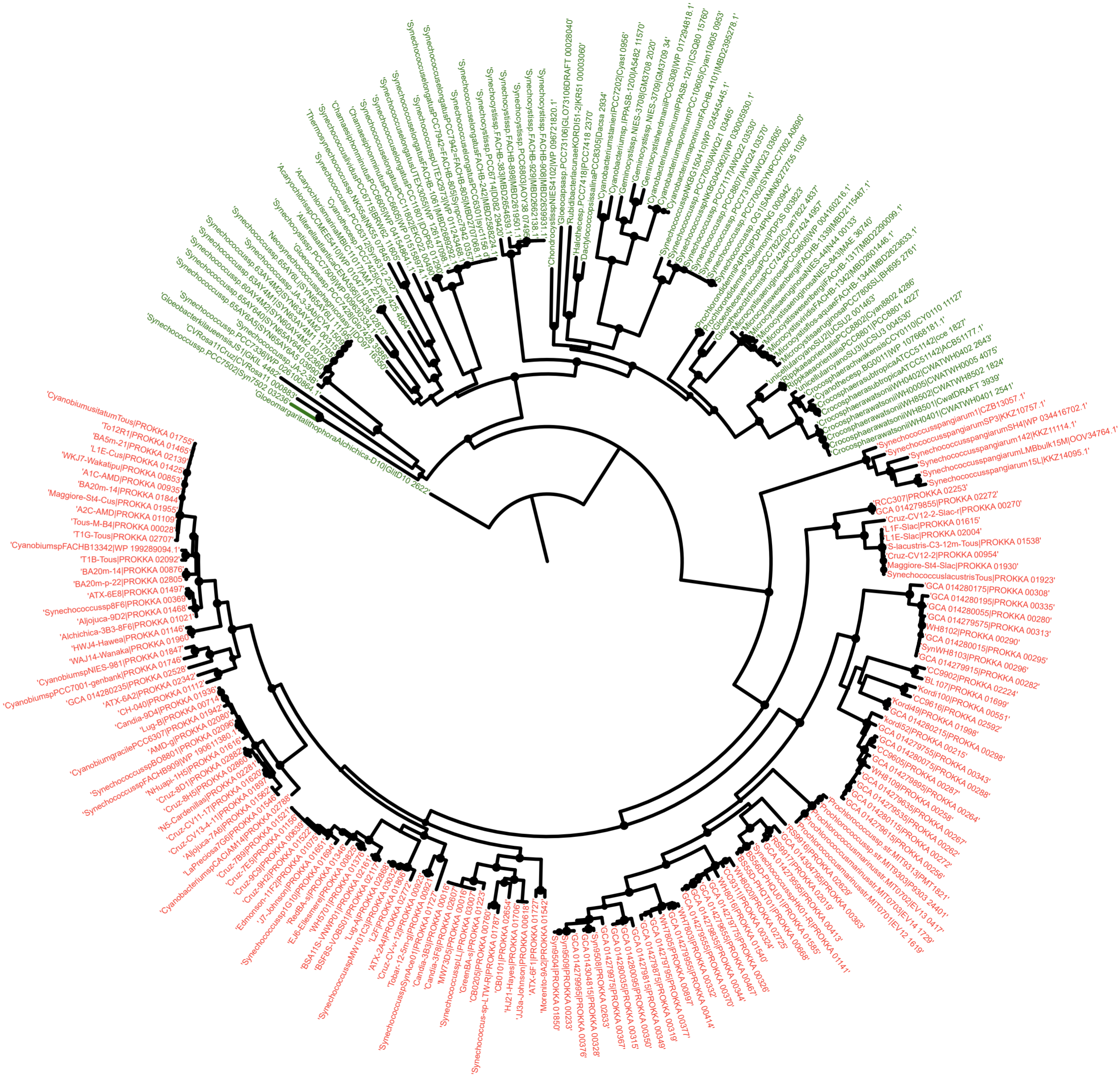

Supplement: Supplementary file 12 — Figure S11 [file 41396_2022_1282_MOESM12_ESM.pdf]

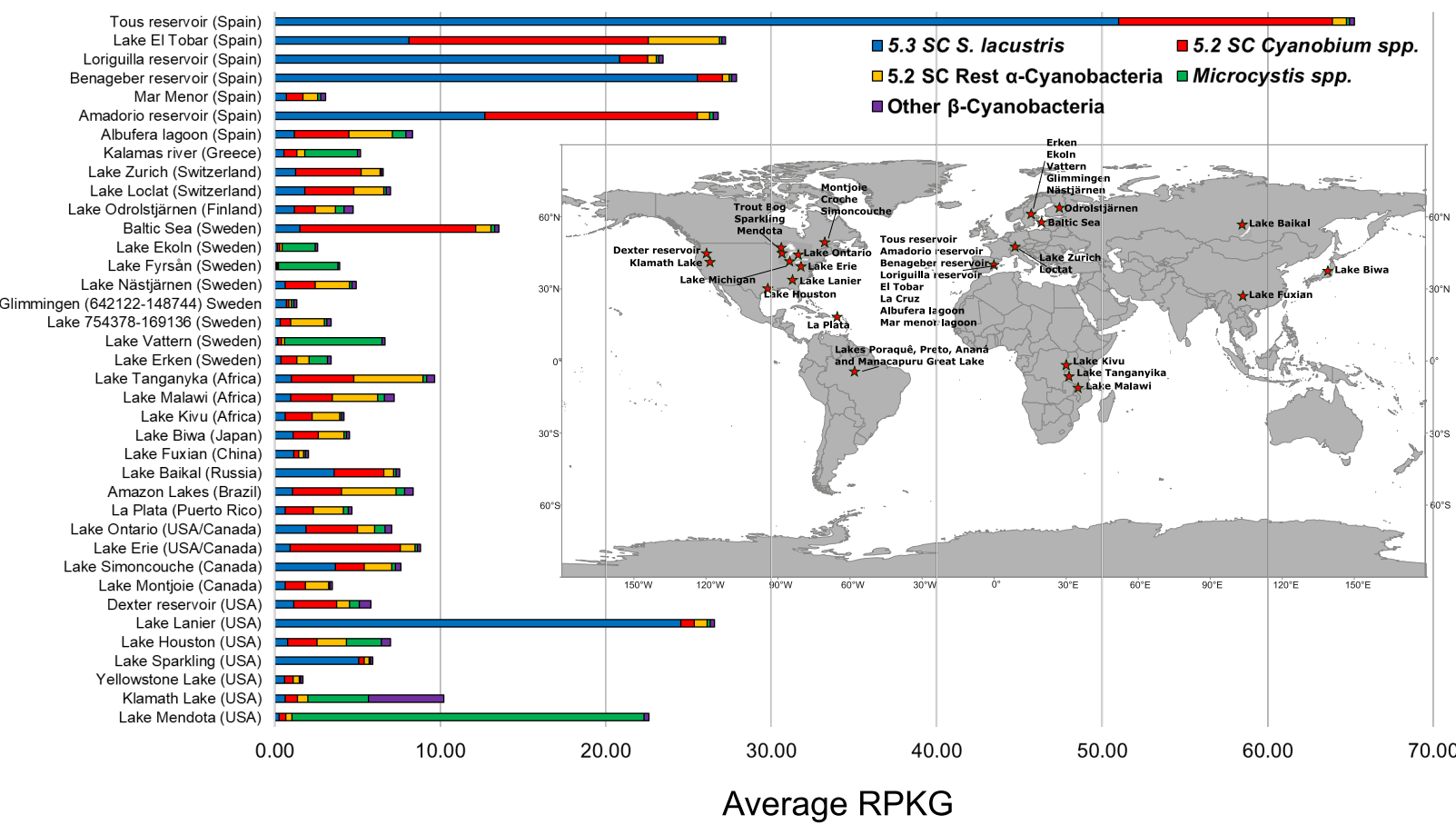

Supplement: Supplementary file 14 — Figure S13 [file 41396_2022_1282_MOESM14_ESM.pdf]

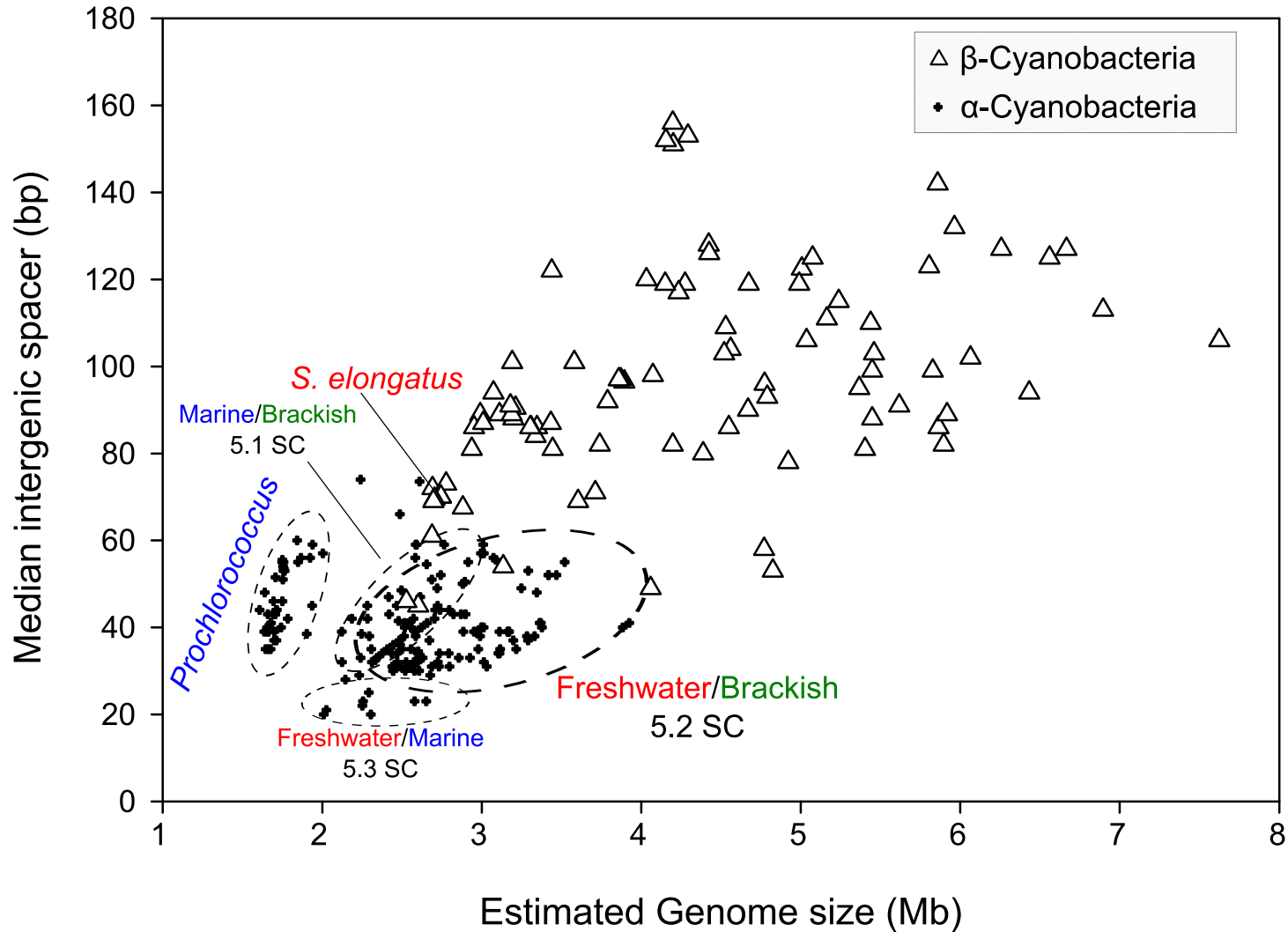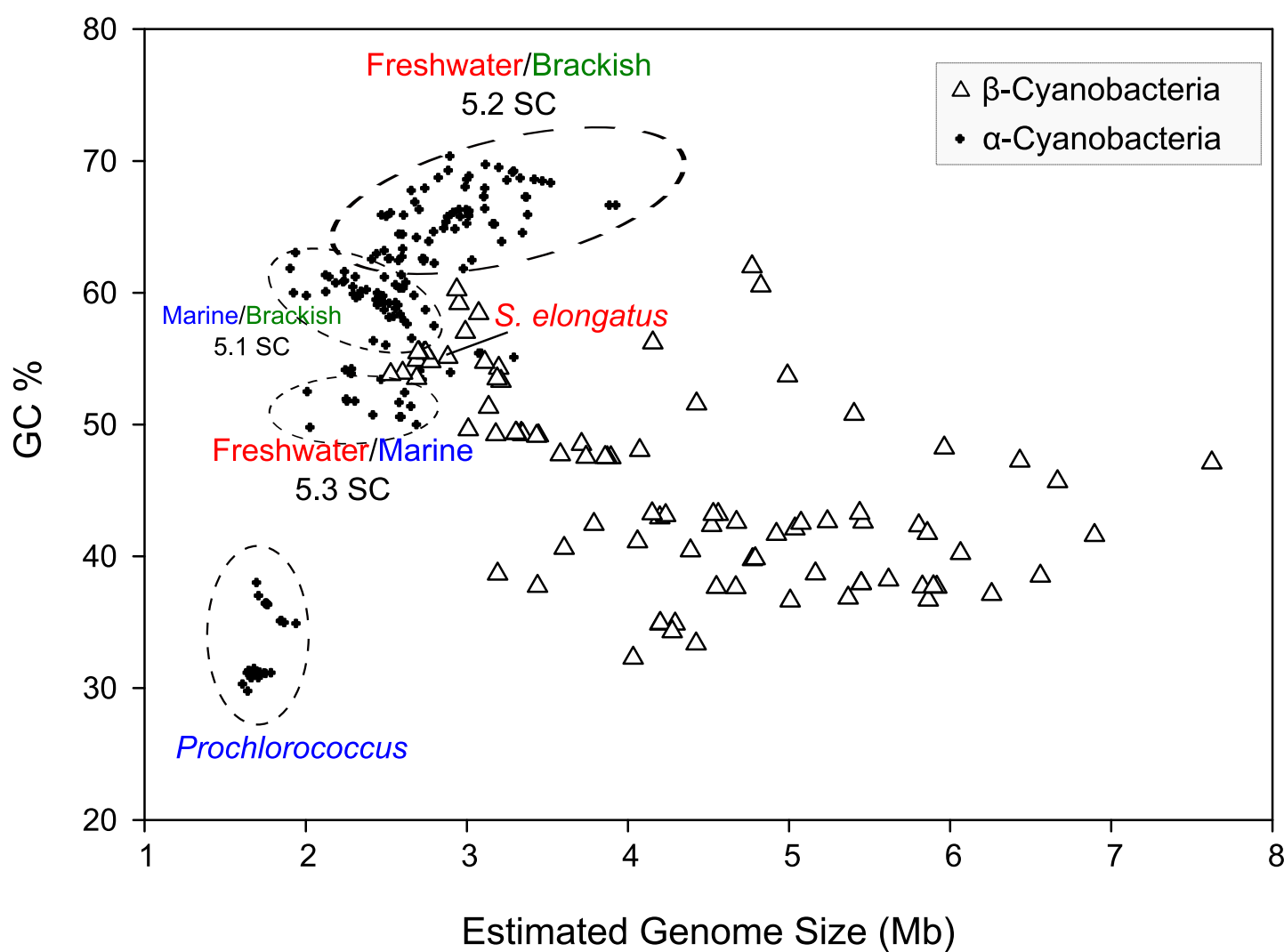

Supplement: Supplementary file 15 — Figure S14 [file 41396_2022_1282_MOESM15_ESM.pdf]
