# Supplementary material for: α-cyanobacteria possessing form IA RuBisCO globally dominate aquatic habitats
Source: ISME J. 2022 Jul 18;16(10):2421–32. doi: 10.1038/s41396-022-01282-z (PMC9477826; doi:10.1038/s41396-022-01282-z)
Supplement: Supplementary file 9 — Figure S8 [file 41396_2022_1282_MOESM9_ESM.pdf]

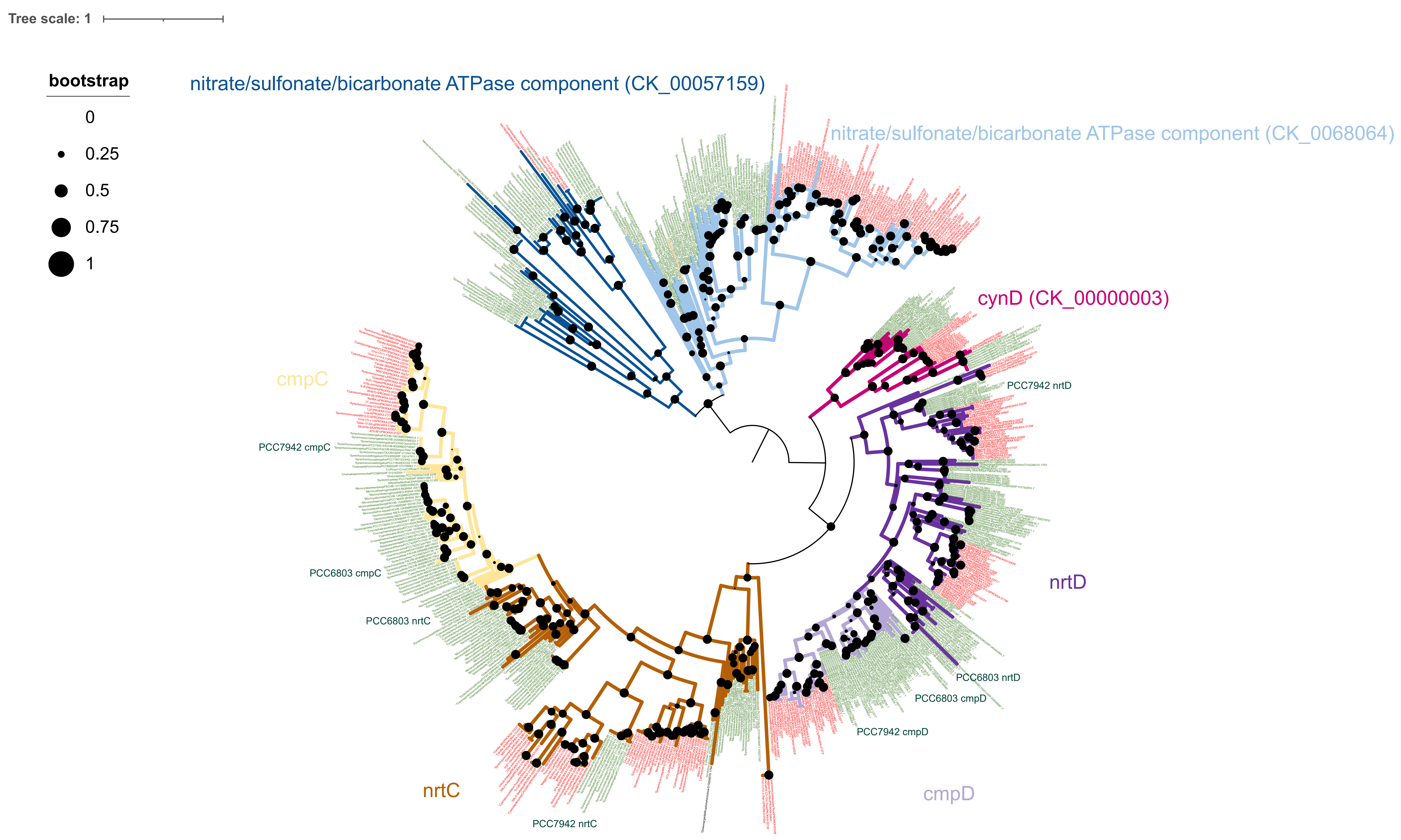

Tree scale: 1
bootstrap
0
0.25
0.5
0.75
1
nitrate/sulfonate/bicarbonate ATPase component (CK_00057159)
nitrate/sulfonate/bicarbonate ATPase component (CK_0068064)
cynD (CK_00000003)
PCC7942 nrtD
nrtD
PCC6803 nrtD
PCC6803 cmpD
PCC7942 cmpD
cmpD
PCC7942 nrtC
nrtC
PCC6803 nrtC
PCC6803 cmpC
PCC7942 cmpC
cmpC
