# Supplementary material for: α-cyanobacteria possessing form IA RuBisCO globally dominate aquatic habitats
Source: ISME J. 2022 Jul 18;16(10):2421–32. doi: 10.1038/s41396-022-01282-z (PMC9477826; doi:10.1038/s41396-022-01282-z)
Supplement: Supplementary file 10 — Figure S9 [file 41396_2022_1282_MOESM10_ESM.pdf]

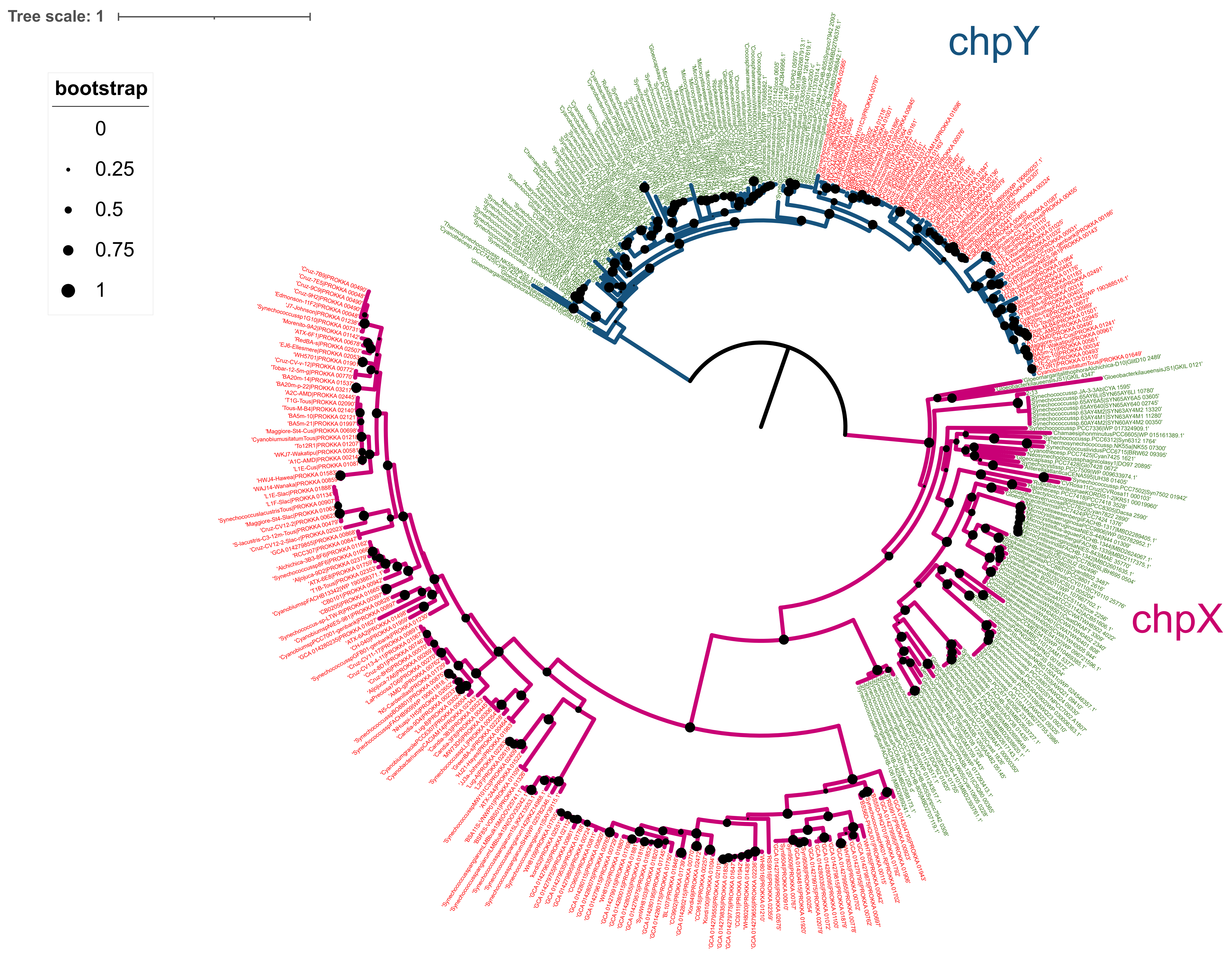
Tree scale: 1
bootstrap
0
0.25
0.5
0.75
1
chpY
chpX
