# Supplementary material for: α-cyanobacteria possessing form IA RuBisCO globally dominate aquatic habitats
Source: ISME J. 2022 Jul 18;16(10):2421–32. doi: 10.1038/s41396-022-01282-z (PMC9477826; doi:10.1038/s41396-022-01282-z)
Supplement: Supplementary file 13 — Figure S12 [file 41396_2022_1282_MOESM13_ESM.pdf]

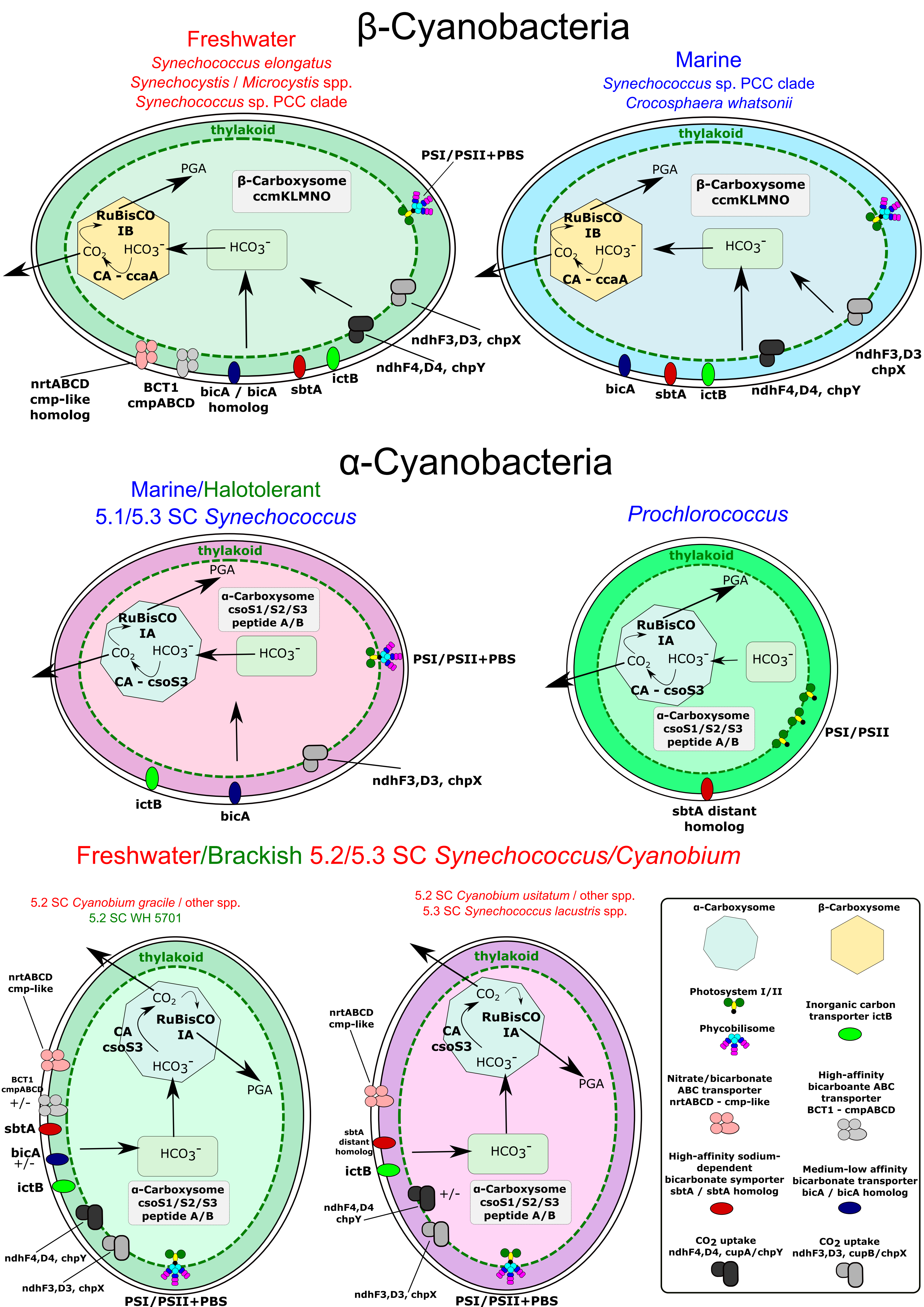
β-Cyanobacteria
Freshwater
Synechococcus elongatus
Synechocystis / Microcystis spp.
Synechococcus sp. PCC clade
thylakoid
PGA
β-Carboxysome
ccmKLMNO
RuBisCO
IB
CO2
HCO3-
CA - ccaA
HCO3-
PSI/PSII+PBS
ndhF3,D3, chpX
ndhF4,D4, chpY
nrtABCD
cmp-like
homolog
BCT1
cmpABCD
bicA / bicA
homolog
sbtA
ictB
Marine
Synechococcus sp. PCC clade
Crocosphaera whatsonii
thylakoid
PGA
β-Carboxysome
ccmKLMNO
RuBisCO
IB
CO2
HCO3-
CA - ccaA
HCO3-
ndhF3,D3
chpX
bicA
sbtA
ictB
ndhF4,D4, chpY
α-Cyanobacteria
Marine/Halotolerant
5.1/5.3 SC Synechococcus
thylakoid
PGA
α-Carboxysome
csoS1/S2/S3
peptide A/B
RuBisCO
IA
CO2
HCO3-
CA - csoS3
HCO3-
PSI/PSII+PBS
ndhF3,D3, chpX
ictB
bicA
Prochlorococcus
thylakoid
PGA
RuBisCO
IA
CO2
HCO3-
CA - csoS3
HCO3-
α-Carboxysome
csoS1/S2/S3
peptide A/B
PSI/PSII
sbtA distant
homolog
Freshwater/Brackish 5.2/5.3 SC Synechococcus/Cyanobium
5.2 SC Cyanobium gracile / other spp.
5.2 SC WH 5701
thylakoid
CO2
RuBisCO
IA
CA
csoS3
HCO3-
PGA
HCO3-
α-Carboxysome
csoS1/S2/S3
peptide A/B
nrtABCD
cmp-like
BCT1
cmpABCD
+/-
sbtA
bicA
+/-
ictB
ndhF4,D4, chpY
ndhF3,D3, chpX
PSI/PSII+PBS
5.2 SC Cyanobium usitatum / other spp.
5.3 SC Synechococcus lacustris spp.
thylakoid
CO2
RuBisCO
IA
CA
csoS3
HCO3-
PGA
HCO3-
α-Carboxysome
csoS1/S2/S3
peptide A/B
nrtABCD
cmp-like
sbtA
distant
homolog
ictB
+/-
ndhF4,D4
chpY
ndhF3,D3, chpX
PSI/PSII+PBS
α-Carboxysome
β-Carboxysome
Photosystem I/II
Phycobilisome
Inorganic carbon
transporter ictB
Nitrate/bicarbonate
ABC transporter
nrtABCD - cmp-like
High-affinity
bicarboante ABC
transporter
BCT1 - cmpABCD
High-affinity sodium-
dependent
bicarbonate symporter
sbtA / sbtA homolog
Medium-low affinity
bicarbonate transporter
bicA / bicA homolog
CO2 uptake
ndhF4,D4, cupA/chpY
CO2 uptake
ndhF3,D3, cupB/chpX
