# Supplementary material for: α-cyanobacteria possessing form IA RuBisCO globally dominate aquatic habitats
Source: ISME J. 2022 Jul 18;16(10):2421–32. doi: 10.1038/s41396-022-01282-z (PMC9477826; doi:10.1038/s41396-022-01282-z)
Supplement: Supplementary file 17 — Table S1 [file 41396_2022_1282_MOESM17_ESM.docx]

**Table S1.** Key features of previously and 58 newly sequenced cluster 5 freshwater picocyanobacteria.

| **Isolate** | **Genome size (bp)** | **GC (%)** | **nº of contigs** | **SC** | **Depth (m)** | **Aquatic system origin** | **Pigment type** |
| --- | --- | --- | --- | --- | --- | --- | --- |
| Lug-A | 3.01 | 66.3 | 33 | 5.2 | - | Lake Lugano, Switzerland/Italy | PC |
| Lug-B | 3.18 | 69.2 | 115 | 5.2 | - | Lake Lugano, Switzerland/Italy | PC |
| La Preciosa 7G6 | 3.13 | 67.3 | 34 | 5.2 | 5 | Lake La Preciosa, Mexico | PE |
| A1C-AMD | 2.46 | 63.2 | 132 | 5.2 | 10 | Amadorio reservoir, Spain | PE |
| A2C-AMD | 3.01 | 62.5 | 112 | 5.2 | 20 | Amadorio reservoir, Spain | PE |
| Aljojuca 7A6 | 3.13 | 67.3 | 34 | 5.2 | 10 | Lake Aljojuca, Mexico | PE |
| AMD-g | 3.12 | 67.9 | 13 | 5.2 | 10 | Amadorio reservoir, Spain | PC |
| ATX 2A4 | 2.92 | 66.1 | 46 | 5.2 | 1 | Lake Atexcac, Mexico | PE |
| ATX 6A2 | 3.01 | 68.0 | 42 | 5.2 | 2 | Lake Atexcac, Mexico | PE |
| ATX 6F1 | 2.96 | 66.3 | 17 | 5.2 | 15 | Lake Atexcac, Mexico | PE |
| BA20m-14 | 2.78 | 64.6 | 228 | 5.2 | 20 | Lake Baikal, Russia | PE |
| BA20m-p-22 | 3.15 | 63.9 | 288 | 5.2 | 20 | Lake Baikal, Russia | PE |
| BA5m-10 | 2.87 | 61.8 | 93 | 5.2 | 5 | Lake Baikal, Russia | PE |
| BA5m-21 | 2.58 | 62.8 | 92 | 5.2 | 5 | Lake Baikal, Russia | PE |
| C3-12m-Tous | 2.57 | 51.7 | 88 | 5.3 | 12 | Tous reservoir, Spain | PE |
| CH-040 | 2.90 | 69.3 | 27 | 5.2 | - | Lake Chascomús, Argentina | PC |
| Cruz-7E5 | 3.16 | 65.2 | 31 | 5.2 | 0.5 | Lake La Cruz, Spain | PC |
| Cruz-8D1 | 3.97 | 66.6 | 108 | 5.2 | 8 | Lake La Cruz, Spain | PC |
| Cruz-8H5 | 3.95 | 66.6 | 92 | 5.2 | 8 | Lake La Cruz, Spain | PC |
| Cruz-9C9 | 3.15 | 65.2 | 33 | 5.2 | 12 | Lake La Cruz, Spain | PC |
| Cruz-9H2 | 3.16 | 65.2 | 33 | 5.2 | 12 | Lake La Cruz, Spain | PC |
| Cruz CV11-17 | 3.42 | 67.3 | 57 | 5.2 | 11 | Lake La Cruz, Spain | PC |
| Cruz CV12-2 | 1.96 | 52.5 | 219 | 5.3 | 12 | Lake La Cruz, Spain | PE |
| Cruz CV12-2-Slac-r | 1.93 | 49.8 | 242 | 5.3 | 12 | Lake La Cruz, Spain | PE |
| Cruz CV13-4-11 | 3.41 | 67.3 | 52 | 5.2 | 13 | Lake La Cruz, Spain | PC |
| Cruz CV-v-12-Cruz | 3.02 | 65.9 | 52 | 5.2 | 12 | Lake La Cruz, Spain | PC |
| N.Huapi 1H5 | 3.14 | 69.7 | 12 | 5.2 | 70 | Nahuel Huapi, Argentina | PC |
| Albano 3B3 | 3.44 | 68.6 | 85 | 5.2 | 0.5 | Lake Albano, Italy | PE |
| Alchichica 3B3-8F6 | 2.48 | 65.9 | 34 | 5.2 | - | Lake Alchichica, Mexico | PE |
| Candia 3F8 | 3.50 | 68.5 | 90 | 5.2 | - | Lake Candia, Italy | PE |
| ATX 6E8 | 2.62 | 65.9 | 35 | 5.2 | 10 | Lake Atexcac, Mexico | PE |
| Morenito 9A2 | 3.10 | 66.4 | 26 | 5.2 | - | Lake Morenito, Argentina | PC |
| Aljojuca 7D2 | 2.54 | 66.1 | 23 | 5.2 | 15 | Lake Aljojuca, Mexico | PE |
| MW73D5 | 3.45 | 68.6 | 87 | 5.2 | 0.5 | Lake Mondsee, Austria | PE |
| Edmonson 11F2 | 3.15 | 65.2 | 31 | 5.2 | - | Edmonson, Anctartica | PC |
| Candia 9D4 | 3.22 | 69.5 | 16 | 5.2 | 0.5 | Lake Candia, Italy | PE |
| GreenBA-s | 2.85 | 70.4 | 92 | 5.2 | 0.5 | Lake Baikal, Russia | PC |
| HJ21-Hayes | 2.94 | 64.9 | 39 | 5.2 | 0-7.5 | Lake Hayes, New Zealand | PC |
| HWJ4-Hawea | 2.35 | 60.0 | 94 | 5.2 | 0-45 | Lake Hawea, New Zealand | PE |
| J7-Johnson | 3.00 | 65.3 | 68 | 5.2 | 5 | Lake Johnson, New Zealand | PC |
| JJ3a-Johnson | 2.85 | 64.9 | 58 | 5.2 | 5 | Lake Johnson, New Zealand | PE |
| L1E-Cus | 2.60 | 62.5 | 33 | 5.2 | 10 | Loriguilla Reservoir, Spain | PE |
| L1E-Slac | 2.26 | 51.8 | 55 | 5.3 | 10 | Loriguilla Reservoir, Spain | PE |
| L1F-Slac | 2.23 | 51.9 | 47 | 5.3 | 20 | Loriguilla Reservoir, Spain | PE |
| L2F | 3.04 | 66.2 | 10 | 5.2 | 20 | Loriguilla Reservoir, Spain | PC |
| Cruz-7B9 | 3.16 | 65.2 | 32 | 5.2 | 0.5 | Lake La Cruz, Spain | PC |
| Maggiore-St4-Cus | 2.76 | 62.6 | 28 | 5.2 | - | Lake Maggiore, Italy | PE |
| Maggiore-St4-Slac | 2.30 | 51.8 | 38 | 5.2 | - | Lake Maggiore, Italy | PE |
| N5-Cardenillas | 2.59 | 68.9 | 210 | 5.2 | 2 | Lake Cardenillas, Spain | PC |
| RedBA-s | 2.72 | 66.3 | 61 | 5.2 | 0.5 | Lake Baikal, Russia | PE |
| T1B-Tous | 2.71 | 66.9 | 122 | 5.2 | 10 | Tous reservoir, Spain | PE |
| T1G-Tous | 2.78 | 62.4 | 52 | 5.2 | 10 | Tous reservoir, Spain | PE |
| To12R1 | 2.52 | 62.6 | 23 | 5.2 | 12 | Tous reservoir, Spain | PE |
| Tobar12-5m-g | 2.95 | 65.8 | 47 | 5.2 | 12.5 | Lake El Tobar, Spain | PC |
| Tous-M-B4 | 2.74 | 62.6 | 11 | 5.2 | 12 | Tous reservoir, Spain | PE |
| WAJ14-Wanaka | 2.14 | 61.2 | 7 | 5.2 | 0-45 | Lake Wanaka, New Zealand | PE |
| WKJ7-Wakatipu | 2.81 | 62.2 | 61 | 5.2 | 0-45 | Lake Wakatipu, New Zealand | PE |
| EJ6-Ellesmere | 2.89 | 65.9 | 200 | 5.2 | 0.5 | Lake Ellesmere, New Zealand | PC |
| *Cyanobium gracile* PCC6307* | 3.34 | 68.7 | 1 | 5.2 | - | Madison, Wisconsin, USA | PC |
| N.Huapi 1G10* | 3.34 | 64.6 | 84 | 5.2 | 70 | Nahuel Huapi, Argentina | PE |
| Alchichica 8F6* | 2.51 | 65.8 | 84 | 5.2 | 2 | Lake Alchichica, Mexico | PE |
| BO8801* | 3.27 | 69.1 | 47 | 5.2 | - | Lake Constance, Switzerland | PC |
| MW101C3* | 3.03 | 66.1 | 18 | 5.2 | - | Lake Mondsee, Austria | PC |
| CACIAM14* | 3.21 | 68.6 | 71 | 5.2 | 0.5 | Tucurui Power Plant Reservoir, Brazil | PC |
| GFB01* | 2.34 | 67.8 | 125 | 5.2 | - | Lago dos Indios, Brazil | PC |
| *V. limneticus* LL* | 3.55 | 68.4 | 160 | 5.2 | 0.5 | Lake Albano, Italy | PE |
| *C. usitatum* Tous* | 2.52 | 62.6 | 25 | 5.2 | 12 | Tous reservoir, Spain | PE |
| *S. lacustris*Tous* | 2.67 | 51.4 | 305 | 5.3 | 12 | Tous reservoir, Spain | PE |

*Indicates strains sequenced prior to this work. PE-phycoerythrin rich-cells. PC-phycocyanin-rich cells.
